# Supplementary material for: Awareness and utilization of genetic testing among Hispanic and Latino adults living in the US: The Hispanic Community Health Study/Study of Latinos
Source: HGG Adv. 2022 Nov 19;4(1):100160. doi: 10.1016/j.xhgg.2022.100160 (PMC9722474; doi:10.1016/j.xhgg.2022.100160)
Supplement: Document S1. Figures S1–S5 and Tables S1–S6 [file mmc1.pdf]

HGGA, Volume *4*

Supplemental information

Awareness and utilization of genetic testing among Hispanic and Latino adults living in the US: The Hispanic Community Health Study/Study of Latinos

Kurt D. Christensen, Mengran Zhang, Lauren N. Galbraith, Einat Granot-Hershkovitz, Sarah C. Nelson, Sara Gonzalez, Maria Argos, Krista M. Perreira, Martha L. Daviglus, Carmen R. Isasi, Jianwen Cai, Gregory A. Talavera, Carrie L. Blout Zawatsky, Robert C. Green, Rosario Isasi, Robert Kaplan, and Tamar Sofer

Awareness and Utilization of Genetic Testing among Hispanic/Latino Adults Living in the US: The Hispanic Community Health Study/Study of Latinos: Supplemental Information

Table of Contents

Figure S1. Spanish version of the genetic testing awareness survey. 2

Figure S2. English version of the genetic testing awareness survey. 4

Figure S3: Proportion of aware participants who were offered and used genetic tests. 6

Figure S4: Awareness, offers, and usage of genetic tests, stratified by gender. 7

Figure S5. Awareness of genetic tests, by demographic factors. 8

Table S1. Characteristics of survey participants. 10

Table S2. Logistic regression models for awareness of genetic tests, by site. 12

Table S3. Logistic regression models for awareness of genetic tests, by gender. 14

Table S4. Logistic regression models about awareness of specific types of genetic tests. 16

Table S5. Poisson regression models of perceived utility, by site. 18

Table S6. Poisson regression models of perceived utility, by gender. 20

Figure S1. Spanish version of the genetic testing awareness survey.

HCHS/SOL- Follow Up Interview

Genetic Testing Awareness, GTS

ID NUMBER:									FORM CODE: GTS VERSION: 1, 4/8/2019	Contact Occasion	0	1	Occurrence	0	1

ADMINISTRATIVE INFORMATION

0a. Completion Date: ☐☐/☐☐/☐☐☐☐ 0b. Staff ID: ☐☐☐

Instructions: Enter the answer given by the participant for each response. Use the CDART Notelog window to code 'Don't know/refused, Missing, etc.' for those questions that do not list these as an option.

INTRODUCTION: Los médicos utilizan estudios genéticos para analizar los genes de las personas por razones de salud.

1. ¿Ha oído usted hablar sobre una prueba genética para determinar el riesgo o la probabilidad de contraer una enfermedad en particular?

   No 0 ☐ **[Go to item 1c]** Sí 1 ☐ Rehusa contestar 9 ☐ **[Go to item 2]**

   1a. ¿Le han ofrecido alguna vez este tipo de prueba?

   No 0 ☐ **[Go to item 1c]** Sí 1 ☐ No sé 8 ☐**[Go to item 1c]** Rehusa contestar 9 ☐

   1b. ¿Le han hecho alguna vez este tipo de prueba?

   No 0 ☐ Sí 1 ☐ **[Go to item 2]** No sé 8 ☐ Rehusa contestar 9 ☐

   1c. Si se la ofrecieran, ¿estaría usted interesado(a) en hacerse este tipo de prueba?

   No 0 ☐ Sí 1 ☐ No estoy seguro(a)/depende 2 ☐ Rehusa contestar 9 ☐

2. ¿Ha oído usted hablar sobre una prueba genética para determinar la probabilidad de transmitir una enfermedad hereditaria a sus hijos?

   No 0 ☐ **[Go to item 2c]** Sí 1 ☐ Rehusa contestar 9 ☐ **[Go to item 3]**

   2a. ¿Le han ofrecido alguna vez este tipo de prueba?

   No 0 ☐ **[Go to item 2c]** Sí 1 ☐ No sé 8 ☐**[Go to item 2c]** Rehusa contestar 9 ☐

   2b. ¿Le han hecho alguna vez este tipo de prueba?

   No 0 ☐ Sí 1 ☐ **[Go to item 3]** No sé 8 ☐ Rehusa contestar 9 ☐

   2c. Si se la ofrecieran, ¿estaría usted interesado(a) en hacerse este tipo de prueba?

   No 0 ☐ Sí 1 ☐ No estoy seguro(a)/depende 2 ☐ Rehusa contestar 9 ☐

3. ¿Ha oído usted hablar sobre una prueba genética para determinar cómo se debe tratar una enfermedad después del diagnóstico?

   No 0 ☐ **[Go to item 3c]** Sí 1 ☐ Rehusa contestar 9 ☐ **[Go to item 4]**

   3a. ¿Le han ofrecido alguna vez este tipo de prueba?

   No 0 ☐ **[Go to item 3c]** Sí 1 ☐ No sé 8 ☐**[Go to item 3c]** Rehusa contestar 9 ☐

   3b. ¿Le han hecho alguna vez este tipo de prueba?

   No 0 ☐ Sí 1 ☐ **[Go to item 4]** No sé 8 ☐ Rehusa contestar 9 ☐

   3c. Si se la ofrecieran, ¿estaría usted interesado(a) en hacerse este tipo de prueba?

   No 0 ☐ Sí 1 ☐ No estoy seguro(a)/depende 2 ☐ Rehusa contestar 9 ☐

Figure S1 (continued).

ID NUMBER:									FORM CODE: GTS VERSION: 1, 4/8/2019	Contact Occasion			Occurrence		

4. ¿Ha oído usted hablar sobre una prueba genética para determinar los medicamentos que pueden servirle o no a una persona (individuo)?

   No 0 ☐ **[Go to item 4c]** Sí 1 ☐ Rehusa contestar 9 ☐ **[Go to item 5]**

   4a. ¿Le han ofrecido alguna vez este tipo de prueba?

   No 0 ☐ **[Go to item 4c]** Sí 1 ☐ No sé 8 ☐**[Go to item 4c]** Rehusa contestar 9 ☐

   4b. ¿Le han hecho alguna vez este tipo de prueba?

   No 0 ☐ Sí 1 ☐ **[Go to item 5]** No sé 8 ☐ Rehusa contestar 9 ☐

   4c. Si se la ofrecieran, ¿estaría usted interesado(a) en hacerse este tipo de prueba?
   No 0 ☐ Sí 1 ☐ No estoy seguro(a)/depende 2 ☐ Rehusa contestar 9 ☐

5. En una escala del 1 al 10, donde 1 es "de ningún modo" y 10 es "absolutamente", ¿qué tan útil cree usted que es la prueba genética para controlar la salud de una persona? ☐☐

Figure S2. English version of the genetic testing awareness survey.

HCHS/SOL- Follow Up Interview

Genetic Testing Awareness, GTE

ID NUMBER:									FORM CODE: GTE<br>VERSION: 1, 4/8/2019	Contact Occasion	0	1	Occurrence	0	1

ADMINISTRATIVE INFORMATION

0a. Completion Date: ☐☐/☐☐/☐☐☐☐ 0b. Staff ID: ☐☐☐

Instructions: Enter the answer given by the participant for each response. Use the CDART Notelog window to code 'Don't know/refused, Missing, etc.' for those questions that do not list these as an option.

INTRODUCTION: Doctors use genetic tests to analyze someone's genes for health reasons.

1. Have you heard of a genetic test to determine the risk or likelihood of getting a particular disease?

   No 0 ☐ **[Go to item 1c]** Yes 1 ☐ Refuse to answer 9 ☐ **[Go to item 2]**

   1a. Have you ever been offered such test?

   No 0 ☐ **[Go to item 1c]** Yes 1 ☐ Don't know 8 ☐ **[Go to item 1c]** Refuse to answer 9 ☐

   1b. Have you ever received this kind of test?

   No 0 ☐ Yes 1 ☐ **[Go to item 2]** Don't know 8 ☐ Refuse to answer 9 ☐

   1c. If offered to you, would you be interested in receiving this kind of test?

   No 0 ☐ Yes 1 ☐ Not sure / it depends 2 ☐ Refuse to answer 9 ☐

2. Have you heard of a genetic test to determine the likelihood of passing an inherited disease to your children?

   No 0 ☐ **[Go to item 2c]** Yes 1 ☐ Refuse to answer 9 ☐ **[Go to item 3]**

   2a. Have you ever been offered such test?

   No 0 ☐ **[Go to item 2c]** Yes 1 ☐ Don't know 8 ☐ **[Go to item 2c]** Refuse to answer 9 ☐

   2b. Have you ever received this kind of test?

   No 0 ☐ Yes 1 ☐ **[Go to item 3]** Don't know 8 ☐ Refuse to answer 9 ☐

   2c. If offered to you, would you be interested in receiving this kind of test?

   No 0 ☐ Yes 1 ☐ Not sure / it depends 2 ☐ Refuse to answer 9 ☐

3. Have you heard of a genetic test to determine how a disease should be treated after diagnosis?

   No 0 ☐ **[Go to item 3c]** Yes 1 ☐ Refuse to answer 9 ☐ **[Go to item 4]**

   3a. Have you ever been offered such test?

   No 0 ☐ **[Go to item 3c]** Yes 1 ☐ Don't know 8 ☐ **[Go to item 3c]** Refuse to answer 9 ☐

   3b. Have you ever received this kind of test?

   No 0 ☐ Yes 1 ☐ **[Go to item 4]** Don't know 8 ☐ Refuse to answer 9 ☐

   3c. If offered to you, would you be interested in receiving this kind of test?
   No 0 ☐ Yes 1 ☐ Not sure / it depends 2 ☐ Refuse to answer 9 ☐

Figure S2 (continued).

ID NUMBER:									FORM CODE: GTE<br>VERSION: 1, 4/8/2019	Contact Occasion			Occurrence		

4. Have you heard of a genetic test to determine which drug(s) may or may not work for an individual?

   No 0 ☐ **[Go to item 4c]** Yes 1 ☐ Refuse to answer 9 ☐**[Go to item 5]**

   4a. Have you ever been offered such test?

   No 0 ☐ **[Go to item 4c]** Yes 1 ☐ Don't know 8 ☐**[Go to item 4c]** Refuse to answer 9 ☐

   4b. Have you ever received this kind of test?

   No 0 ☐ Yes 1 ☐ **[Go to item 5]** Don't know 8 ☐ Refuse to answer 9 ☐

   4c. If offered to you, would you be interested in receiving this kind of test?

   No 0 ☐ Yes 1 ☐ Not sure / it depends 2 ☐ Refuse to answer 9 ☐

5. On a scale of 1 to 10, where 1 is "not at all" and 10 is "extremely", how useful do you think genetic testing is for managing a person's health? ☐☐

Figure S3: Proportion of aware participants who were offered and used genetic tests.

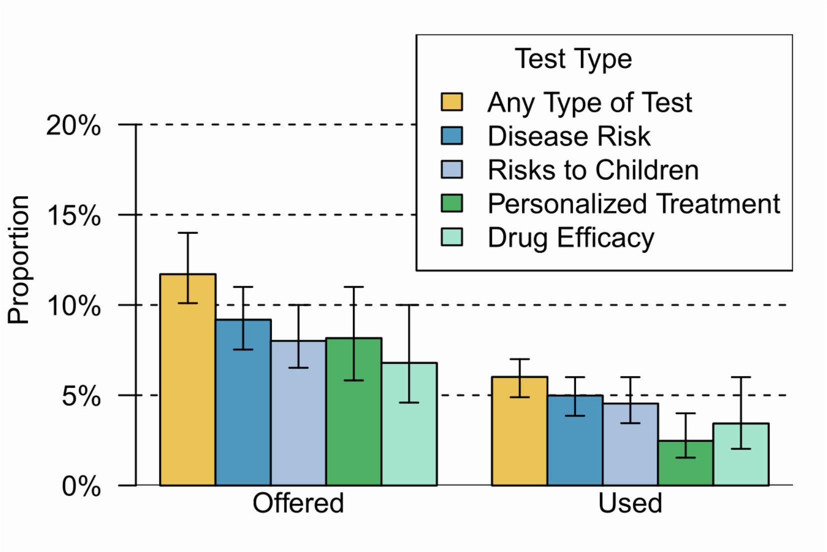

Bars represent estimates and 95% CIs of the proportions of participants who were aware of genetic tests that additionally reported ever being offered or using them.

Figure S4: Awareness, offers, and usage of genetic tests, stratified by gender.

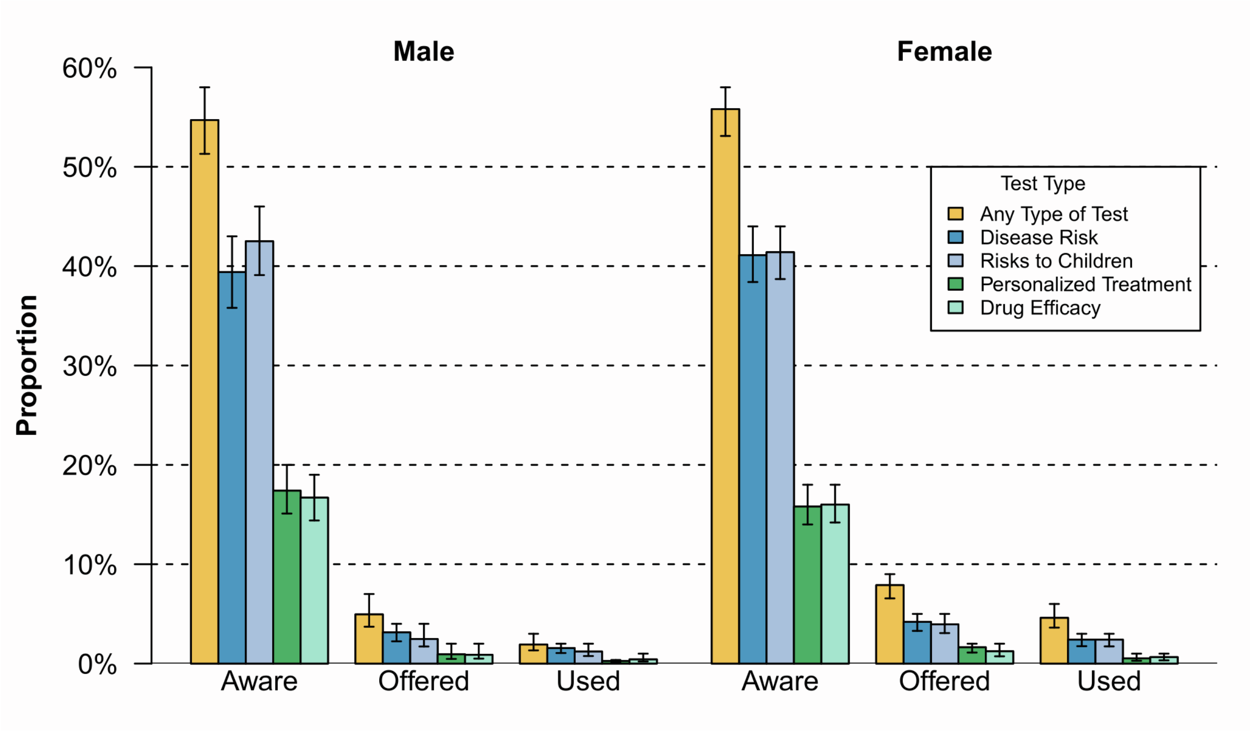

Bars represent estimates and 95% CIs of the proportions of participants who reported being aware of genetic tests, being offered them, or using them, stratified by gender.

Figure S5. Awareness of genetic tests, by demographic factors.

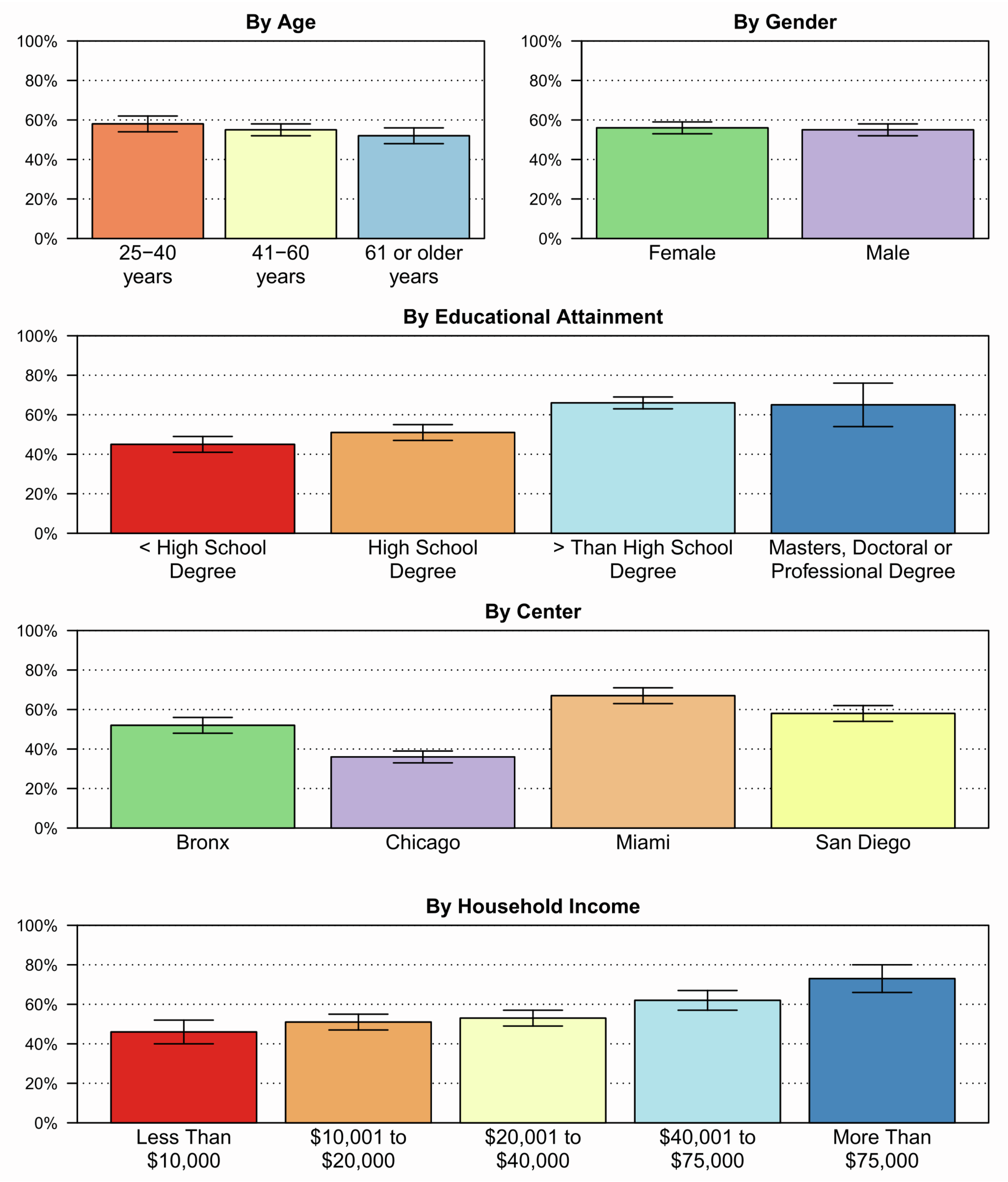

Figure S5 (continued)

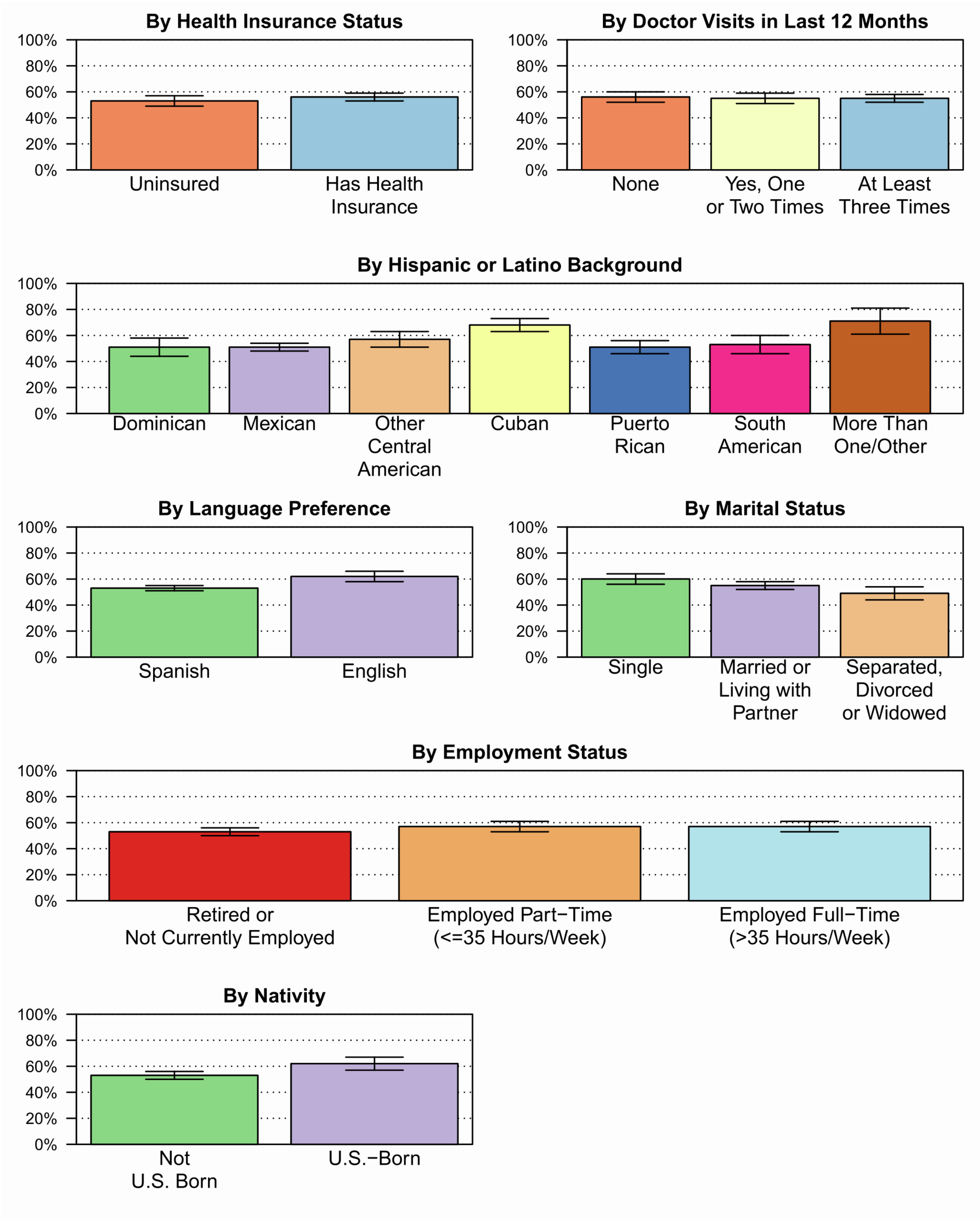

Bars represent estimates and 95% CIs of the proportions of participants who reported being aware of at least one type of genetic test, stratified by demographic factors of interest.

Table S1. Characteristics of survey participants.

Total N	Did not Complete Any Awareness Items (n=3,639)	Completed at Least One Awareness Item (n=5,769)	p	% Missing
Mean Age, years	46.31 (15.25)	45.81 (14.55)	0.081	0
Sex			0.023	0
Female	2249 (51.1)	3701 (53.3)		
Male	1390 (48.9)	2068 (46.7)		
Center			<0.001	0
Bronx	1357 (41.9)	1030 (21.7)		
Chicago	658 (9.0)	1950 (20.4)		
Miami	736 (30.4)	1065 (27.6)		
San Diego	888 (18.7)	1724 (30.4)		
Education			<0.001	0.5
Less than high school degree	1464 (36.9)	2004 (28.8)		
High school degree	946 (28.0)	1458 (26.9)		
Associate, bachelor, or vocational degree	1103 (32.9)	2078 (40.4)		
Masters, doctoral, professional degree	95 (2.1)	206 (4.0)		
Household Income			<0.001	3.1
Less than $10,000	486 (14.5)	613 (9.7)		
$10,001-$20,000	1057 (28.7)	1532 (25.7)		
$20,001-$40,000	1162 (32.8)	1999 (34.4)		
$40,001-$75,000	571 (17.2)	1039 (19.7)		
More than $75,000	214 (6.7)	441 (10.5)		
Health Insurance Status			0.690	0.7
Uninsured	1037 (30.9)	1686 (31.5)		
Has health insurance	2552 (69.1)	4068 (68.5)		
Doctor Visit in Last 12 Months			0.824	1.8

No	944 (31.1)	1521 (32.0)		
Yes, one or two times	1113 (30.9)	1820 (30.2)		
Yes, at least three times	1484 (38.0)	2352 (37.8)		
Hispanic or Latino background			<0.001	0.5
Dominican	449 (13.6)	413 (8.1)		
Mexican	1344 (29.4)	2753 (42.4)		
Central American	332 (7.7)	575 (7.0)		
Cuban	426 (21.2)	593 (19.2)		
Puerto Rican	717 (19.1)	820 (14.4)		
South American	228 (4.7)	424 (5.2)		
More than one/other	112 (4.4)	172 (3.7)		
Language Preference			<0.001	0
Spanish	2859 (73.2)	4731 (75.7)		
English	780 (26.8)	1038 (24.3)		
Marital Status			<0.001	0.3
Single	906 (33.1)	1234 (29.0)		
Married or living with partner	1935 (48.1)	3288 (53.5)		
Separated, divorced or widowed	777 (18.8)	1240 (17.5)		
Employment Status			<0.001	0.7
Retired/not currently employed	1660 (44.5)	2362 (37.4)		
Employed part-time (≤35 hours/week)	730 (20.1)	1318 (22.2)		
Employed full-time (>35 hours/week)	1199 (35.3)	2071 (40.4)		
Nativity				
Not U.S. Born	2969 (77.0)	4835 (77.5)	0.021	0.5
U.S.-Born	643 (23.0)	918 (22.5)		

The table summarizes characteristics of HCHS/SOL participants who participated in the annual follow-up survey. Analyses compare the characteristics of individuals who completed at least one genetic testing awareness item against characteristics of individuals who did not.

Table S2. Logistic regression models for awareness of genetic tests, by site.

	Bronx	Chicago	Miami	San Diego
**Term**	**OR (95% CI)**	**OR (95% CI)**	**OR (95% CI)**	**OR (95% CI)**
Age, years (ref: 40 and younger)				
41-60	0.93 (0.60-1.43)	1.07 (0.81-1.41)	0.89 (0.51-1.54)	1.14 (0.76-1.71)
61 or older	1.34 (0.77-2.33)	0.94 (0.60-1.46)	0.66 (0.34-1.28)	0.96 (0.47-1.94)
Male sex (ref: Female)	0.81 (0.54-1.21)	1.01 (0.76-1.35)	0.86 (0.60-1.22)	0.87 (0.60-1.25)
Education, years (ref: less than high school degree)				
High school degree	0.76 (0.47-1.21)	1.61 (1.16-2.22)*	1.16 (0.68-1.99)	1.02 (0.65-1.58)
Associate, bachelor, or vocational degree	1.90 (1.23-2.94)*	2.26 (1.56-3.27)**	1.44 (0.90-2.29)	1.70 (1.10-2.64)
Masters, doctoral, professional degree	1.46 (0.46-4.65)	3.17 (1.29-7.77)	1.04 (0.49-2.20)	2.80 (1.37-5.74)*
Income (ref: Less than $10,000)				
$10,001-$20,000	1.46 (0.86-2.49)	0.89 (0.51-1.55)	1.24 (0.69-2.24)	0.68 (0.36-1.28)
$20,001-$40,000	1.39 (0.73-2.64)	0.97 (0.58-1.62)	1.09 (0.60-1.98)	1.11 (0.61-2.03)
$40,001-$75,000	1.54 (0.71-3.31)	0.98 (0.54-1.77)	2.40 (1.11-5.22)	1.73 (0.86-3.48)
More than $75,000	1.60 (0.75-3.43)	1.60 (0.79-3.24)	1.87 (0.78-4.53)	3.24 (1.30-8.08)
Health Insurance Status (ref: None)	0.81 (0.41-1.57)	1.19 (0.85-1.67)	0.92 (0.63-1.33)	1.24 (0.82-1.86)
Doctor Visit in Last 12 Months (ref: none)				
Yes, one or two times	1.08 (0.57-2.03)	1.32 (0.93-1.88)	1.02 (0.65-1.58)	0.86 (0.57-1.30)
At least three times	1.20 (0.64-2.27)	1.37 (0.97-1.92)	0.74 (0.42-1.29)	1.00 (0.69-1.45)

Hispanic or Latino Background (ref: Dominican)				
Mexican	1.07 (0.49-2.33)	0.68 (0.24-1.92)	0.16 (0.02-1.24)	NA†
Central American	1.03 (0.45-2.35)	0.79 (0.25-2.47)	0.77 (0.28-2.10)	NA†
Cuban	4.02 (0.69-23.40)	0.77 (0.21-2.82)	0.96 (0.34-2.65)	NA†
Puerto Rican	1.08 (0.68-1.70)	0.62 (0.22-1.78)	1.14 (0.24-5.48)	NA†
South American	1.57 (0.71-3.45)	0.47 (0.16-1.33)	0.68 (0.21-2.20)	NA†
More than one/other	1.36 (0.52-3.55)	1.32 (0.35-4.98)	2.99 (0.58-15.40)	NA†
English Language Preference (ref: Spanish)	0.91 (0.53-1.56)	1.00 (0.63-1.59)	1.98 (0.72-5.39)	1.65 (1.10-2.48)
Marital Status (ref: Single)				
Married or living with partner	0.69 (0.47-1.02)	1.34 (0.99-1.82)	0.73 (0.44-1.22)	0.89 (0.57-1.40)
Separated, divorced or widowed	0.48 (0.27-0.85)	1.41 (0.89-2.23)	0.66 (0.37-1.20)	0.63 (0.36-1.12)
Employment Status (ref: Retired/not currently employed)				
Employed part-time (≤35 hours/week)	1.51 (0.88-2.59)	0.97 (0.62-1.53)	0.80 (0.49-1.31)	1.17 (0.76-1.78)
Employed full-time (>35 hours/week)	1.16 (0.69-1.93)	0.93 (0.66-1.31)	0.91 (0.58-1.44)	0.91 (0.60-1.39)
U.S.-born (ref: Not U.S.-born)	1.26 (0.71-2.25)	1.11 (0.67-1.84)	1.49 (0.66-3.38)	0.95 (0.61-1.48)

* p<0.01 ** p<0.001

The table summarizes logistic regression analyses that examined associations between participant characteristics and the likelihood of reporting awareness of at least one type of genetic test, with separate models for each HCHS/SOL site of enrollment. Estimates represent odds ratios with 95% confidence intervals, and shows the increase in odds of reporting awareness relative to the reference category. Analyses included all covariates jointly, and were weighted to generate estimates valid for the HCHS/SOL population.

† **Results by Hispanic or Latino background at the San Diego site are omitted because 94% self-reported Mexican heritage.**

Table S3. Logistic regression models for awareness of genetic tests, by gender.

	Women	Men
**Term**	**OR (95% CI)**	**OR (95% CI)**
Age, years (ref: 40 and younger)		
41-60	1.15 (0.87-1.52)	0.86 (0.61-1.22)
61 or older	1.16 (0.82-1.63)	0.69 (0.42-1.14)
Education, years (ref: less than high school degree)		
High school degree	1.03 (0.78-1.35)	1.05 (0.72-1.53)
Associate, bachelor, or vocational degree	1.50 (1.13-1.99)*	1.95 (1.36-2.80)**
Masters, doctoral, professional degree	1.10 (0.61-1.98)	2.07 (0.97-4.39)
Center (ref: Bronx)		
Chicago	0.64 (0.43-0.97)	0.44 (0.26-0.75)*
Miami	1.77 (1.09-2.89)	1.82 (0.98-3.37)
San Diego	1.47 (0.88-2.45)	0.92 (0.48-1.76)
Income (ref: Less than $10,000)		
$10,001-$20,000	1.16 (0.83-1.61)	1.17 (0.63-2.18)
$20,001-$40,000	1.52 (1.11-2.08)*	0.97 (0.52-1.81)
$40,001-$75,000	2.15 (1.42-3.27)**	1.44 (0.76-2.71)
More than $75,000	3.76 (2.20-6.42)**	1.93 (0.95-3.93)
Health Insurance Status (ref: None)	0.93 (0.72-1.20)	1.10 (0.77-1.55)
Doctor Visit in Last 12 Months (ref: none)		
Yes, one or two times	1.03 (0.75-1.43)	0.98 (0.69-1.39)
At least three times	1.06 (0.78-1.45)	0.95 (0.65-1.39)
Hispanic or Latino Background (ref: Dominican)		
Mexican	0.57 (0.32-1.03)	2.04 (0.93-4.47)
Other Central American	0.99 (0.57-1.75)	1.28 (0.57-2.90)
Cuban	0.90 (0.48-1.67)	1.90 (0.78-4.67)
Puerto Rican	0.69 (0.43-1.11)	1.61 (0.80-3.23)

South American	0.86 (0.46-1.62)	1.16 (0.54-2.48)
More than one/other	1.09 (0.44-2.67)	2.27 (0.91-5.70)
English Language Preference (ref: Spanish)	1.27 (0.89-1.80)	1.06 (0.72-1.55)
Marital Status (ref: Single)		
Married or living with partner	0.78 (0.58-1.05)	0.95 (0.68-1.33)
Separated, divorced or widowed	0.66 (0.47-0.95)	0.77 (0.48-1.23)
Employment Status (ref: Retired/not currently employed)		
Employed part-time (≤35 hours/week)	1.02 (0.77-1.36)	1.15 (0.72-1.82)
Employed full-time (>35 hours/week)	1.04 (0.79-1.38)	0.86 (0.58-1.27)
U.S.-born (ref: Not U.S.-born)	1.34 (0.90-1.98)	1.02 (0.65-1.58)

* p<0.01

** p<0.001

The table summarizes logistic regression analyses that examined associations between participant characteristics and the likelihood of reporting awareness of at least one type of genetic test. Estimates represent odds ratios with 95% confidence intervals, and shows the increase in odds of reporting awareness relative to the reference category. Analyses included all covariates jointly, and were weighted to generate estimates valid for the HCHS/SOL population.

Table S4. Logistic regression models about awareness of specific types of genetic tests.

Term	Disease Risk OR (95%CI)	Risks to Children OR (95%CI)	Personalized Treatment OR (95%CI)	Drug Efficacy OR (95%CI)
Age, years (ref: 40 and younger)				
41-60	0.90 (0.71-1.16)	0.89 (0.71-1.12)	1.47 (1.04-2.09)	1.23 (0.90-1.68)
61 or older	0.96 (0.71-1.28)	0.78 (0.59-1.04)	1.34 (0.90-1.99)	1.16 (0.80-1.70)
Male sex (ref: Female)	0.82 (0.67-1.00)	0.91 (0.74-1.11)	1.15 (0.91-1.45)	0.98 (0.77-1.24)
Education, years (ref: less than high school degree)				
High school degree	1.09 (0.85-1.40)	1.14 (0.88-1.48)	1.20 (0.87-1.66)	1.38 (1.01-1.89)
Associate, bachelor, or vocational degree	1.70 (1.34-2.17)**	1.71 (1.36-2.16)**	1.78 (1.30-2.43)**	1.81 (1.35-2.42)**
Masters, doctoral, professional degree	2.01 (1.20-3.38)*	1.93 (1.17-3.19)	2.45 (1.42-4.22)*	3.01 (1.86-4.85)**
Center (ref: Bronx)				
Chicago	0.87 (0.63-1.20)	0.74 (0.51-1.06)	0.94 (0.62-1.43)	1.21 (0.81-1.80)
Miami	3.02 (2.11-4.31)**	1.39 (0.92-2.11)	1.15 (0.74-1.80)	1.17 (0.73-1.87)
San Diego	1.50 (1.03-2.21)	1.19 (0.75-1.89)	0.82 (0.49-1.39)	1.34 (0.84-2.14)
Income (ref: Less than $10,000)				
$10,001-$20,000	1.17 (0.83-1.64)	1.04 (0.76-1.43)	0.97 (0.63-1.49)	1.00 (0.65-1.52)
$20,001-$40,000	1.18 (0.85-1.65)	1.12 (0.81-1.57)	0.89 (0.58-1.34)	0.84 (0.56-1.26)
$40,001-$75,000	1.81 (1.22-2.68)*	1.55 (1.07-2.26)	1.11 (0.70-1.75)	1.02 (0.64-1.62)
More than $75,000	3.44 (2.15-5.51)**	1.84 (1.18-2.85)*	1.55 (0.89-2.72)	1.33 (0.78-2.26)
Has health insurance (ref: does not)	1.05 (0.83-1.33)	0.98 (0.79-1.22)	1.21 (0.93-1.57)	1.03 (0.78-1.36)

Doctor visit (ref: none)				
Yes, one or two times	1.08 (0.85-1.39)	0.98 (0.77-1.24)	0.90 (0.69-1.18)	1.01 (0.76-1.33)
At least three times	0.94 (0.73-1.23)	0.95 (0.74-1.22)	0.86 (0.66-1.13)	0.91 (0.68-1.23)
Heritage (ref: Dominican)				
Mexican	0.65 (0.41-1.03)	0.90 (0.55-1.46)	1.04 (0.57-1.88)	1.09 (0.62-1.93)
Central American	0.64 (0.41-1.00)	1.15 (0.71-1.86)	0.75 (0.42-1.36)	1.01 (0.56-1.82)
Cuban	0.90 (0.56-1.44)	1.28 (0.75-2.17)	0.80 (0.45-1.41)	1.00 (0.53-1.87)
Puerto Rican	0.86 (0.56-1.34)	0.87 (0.60-1.27)	0.90 (0.54-1.52)	1.03 (0.62-1.70)
South American	0.84 (0.50-1.39)	0.98 (0.63-1.52)	0.61 (0.34-1.11)	0.78 (0.42-1.48)
More than one/other	1.02 (0.57-1.83)	1.55 (0.85-2.84)	1.44 (0.72-2.88)	1.52 (0.76-3.06)
English Language Preference (ref: Spanish)	1.25 (0.92-1.69)	1.13 (0.86-1.48)	1.38 (0.97-1.95)	1.39 (1.03-1.87)
Marital Status (ref: Single)				
Separated, divorced or widowed	0.84 (0.65-1.08)	0.91 (0.73-1.13)	1.16 (0.88-1.51)	1.03 (0.78-1.36)
Married or Living with Partner	0.81 (0.60-1.09)	0.78 (0.58-1.05)	0.91 (0.65-1.28)	0.67 (0.47-0.96)
Employment Status (ref: Retired/not currently employed)				
Employed full-time (>35 hours/week)	1.15 (0.89-1.48)	1.05 (0.82-1.34)	0.91 (0.66-1.26)	0.86 (0.62-1.19)
Employed part-time (≤35 hours/week)	0.98 (0.76-1.26)	1.10 (0.88-1.38)	0.67 (0.50-0.91)	0.79 (0.58-1.08)
U.S.-born (ref: Not U.S.-born)	1.05 (0.76-1.45)	1.11 (0.83-1.47)	1.04 (0.72-1.49)	0.93 (0.67-1.29)

* $p<0.01$ ** $p<0.001$

The table summarizes logistic regression analyses that examined associations between participant characteristics and the likelihood of reporting awareness of specific genetic tests. Estimates represent odds ratios with 95% confidence intervals, and shows the increase in odds of reporting awareness relative to the reference category. Analyses included all covariates jointly, and were weighted to generate estimates valid for the HCHS/SOL population.

Table S5. Poisson regression models of perceived utility, by site.

Term	Bronx<br>Estimate (95% CI)	Chicago<br>Estimate (95% CI)	Miami<br>Estimate (95% CI)	San Diego<br>Estimate (95% CI)
Age, years (ref: 40 and younger)				
41-60	1.01 (0.94-1.08)	1.01 (0.96-1.05)	1.05 (1.01-1.09)	1.03 (0.98-1.09)
61 or older	0.91 (0.83-0.99)	0.95 (0.89-1.01)	1.05 (1.00-1.09)	1.01 (0.95-1.06)
Male sex (ref: Female)	0.97 (0.91-1.03)	1.00 (0.97-1.04)	0.99 (0.96-1.02)	0.97 (0.94-1.00)
Education, years (ref: less than high school degree)				
High school degree	1.04 (0.96-1.13)	1.01 (0.97-1.06)	0.99 (0.94-1.03)	1.00 (0.95-1.04)
Associate, bachelor, or vocational degree	1.08 (1.02-1.14)*	1.02 (0.97-1.07)	1.02 (0.97-1.06)	1.00 (0.95-1.05)
Masters, doctoral, professional degree	1.03 (0.90-1.18)	1.03 (0.95-1.11)	0.93 (0.85-1.01)	0.94 (0.86-1.04)
Income (ref: Less than $10,000)				
$10,001-$20,000	1.03 (0.94-1.13)	1.04 (0.97-1.10)	0.99 (0.93-1.06)	0.97 (0.90-1.05)
$20,001-$40,000	1.02 (0.92-1.13)	0.98 (0.93-1.04)	1.03 (0.98-1.08)	0.97 (0.90-1.05)
$40,001-$75,000	1.06 (0.96-1.18)	0.98 (0.91-1.06)	1.03 (0.98-1.08)	1.00 (0.92-1.09)
More than $75,000	0.93 (0.83-1.05)	0.93 (0.84-1.04)	1.01 (0.92-1.09)	0.99 (0.89-1.09)
Health Insurance Status (ref: None)	1.07 (0.98-1.16)	1.01 (0.97-1.05)	0.98 (0.95-1.01)	0.99 (0.95-1.03)
Doctor Visit in Last 12 Months (ref: none)				
Yes, one or two times	0.95 (0.87-1.03)	1.04 (1.00-1.08)	1.01 (0.99-1.04)	1.03 (0.98-1.08)
At least three times	1.00 (0.93-1.08)	1.02 (0.97-1.07)	0.97 (0.95-1.00)	1.02 (0.97-1.07)

Hispanic or Latino Background (ref: Dominican)				
Mexican	1.00 (0.90-1.11)	1.09 (0.87-1.36)	0.89 (0.74-1.07)	0.86 (0.81-0.91)**
Other Central American	1.05 (0.99-1.11)	1.11 (0.88-1.39)	0.93 (0.87-1.00)	0.88 (0.76-1.02)
Cuban	0.89 (0.68-1.17)	1.16 (0.90-1.48)	0.97 (0.91-1.04)	0.92 (0.76-1.11)
Puerto Rican	1.01 (0.95-1.06)	1.14 (0.91-1.43)	0.98 (0.88-1.08)	0.88 (0.76-1.02)
South American	1.03 (0.96-1.11)	1.11 (0.88-1.41)	0.91 (0.86-0.97)*	0.88 (0.73-1.05)
More than one/other	1.07 (0.96-1.18)	1.03 (0.81-1.32)	1.00 (0.92-1.07)	0.79 (0.71-0.88)**
English Language Preference (ref: Spanish)	0.94 (0.89-0.99)	0.97 (0.88-1.06)	1.01 (0.93-1.10)	0.94 (0.89-0.99)
Marital Status (ref: Single)				
Married or living with partner	1.06 (0.99-1.15)	0.95 (0.89-1.01)	1.00 (0.96-1.04)	1.00 (0.96-1.05)
Separated, divorced or widowed	1.09 (1.01-1.17)	0.98 (0.91-1.06)	0.99 (0.95-1.03)	1.01 (0.96-1.07)
Employment Status (ref: Retired/not currently employed)				
Employed part-time (≤35 hours/week)	1.05 (1.00-1.11)	0.99 (0.95-1.04)	1.01 (0.97-1.06)	1.01 (0.97-1.06)
Employed full-time (>35 hours/week)	1.01 (0.94-1.08)	0.99 (0.95-1.04)	1.00 (0.97-1.04)	0.98 (0.94-1.03)
U.S.-born (ref: Not U.S.-born)	0.96 (0.90-1.03)	0.90 (0.83-0.96)*	0.93 (0.87-0.98)	0.97 (0.93-1.01)

* p<0.01 ** p<0.001

The table summarizes Poisson regression analyses that examined associations between participant characteristics and the perceived utility of genetic testing, rated on a 1-10 scale, with separate models for each study site. Estimates represent exponentiated coefficients, and show the increase in perceived utility scores relative to the reference category. Analyses included all covariates jointly, and were weighted to generate estimates valid for the HCHS/SOL population.

Table S6. Poisson regression models of perceived utility, by gender.

Term	Women<br>Estimate (95% CI)	Men<br>Estimate (95% CI)
Age, years (ref: 40 and younger)		
41-60	1.03 (1.00-1.07)	1.01 (0.96-1.07)
61 or older	0.99 (0.95-1.03)	0.97 (0.91-1.03)
Education, years (ref: less than high school degree)		
High school degree	1.03 (1.00-1.06)	0.99 (0.94-1.05)
Associate, bachelor, or vocational degree	1.02 (0.99-1.05)	1.05 (1.00-1.09)
Masters, doctoral, professional degree	1.01 (0.94-1.08)	0.93 (0.85-1.01)
Center (ref: Bronx)		
Chicago	0.97 (0.92-1.02)	1.06 (0.98-1.15)
Miami	1.04 (0.99-1.10)	1.10 (1.02-1.18)
San Diego	0.99 (0.93-1.04)	1.08 (0.98-1.19)
Income (ref: Less than $10,000)		
$10,001-$20,000	1.05 (0.99-1.10)	0.95 (0.86-1.04)
$20,001-$40,000	1.04 (0.99-1.10)	0.95 (0.87-1.03)
$40,001-$75,000	1.06 (1.00-1.12)	0.98 (0.91-1.06)
More than $75,000	1.04 (0.97-1.12)	0.92 (0.84-1.00)
Health Insurance Status (ref: None)	1.01 (0.98-1.04)	0.98 (0.94-1.02)
Doctor Visit in Last 12 Months (ref: none)		
Yes, one or two times	1.00 (0.97-1.04)	1.01 (0.97-1.05)
At least three times	0.98 (0.95-1.02)	1.04 (1.00-1.09)
Hispanic or Latino Background(ref: Dominican)		
Mexican	1.05 (0.99-1.12)	0.92 (0.82-1.03)
Other Central American	1.02 (0.97-1.08)	0.97 (0.90-1.05)
Cuban	1.04 (0.98-1.11)	0.99 (0.91-1.09)

Puerto Rican	1.00 (0.93-1.07)	0.97 (0.89-1.06)
South American	1.02 (0.97-1.08)	0.94 (0.85-1.04)
More than one/other	1.06 (0.98-1.14)	0.89 (0.80-0.99)
English Language Preference (ref: Spanish)	0.97 (0.93-1.01)	0.93 (0.88-0.98)*
Marital Status (ref: Single)		
Married or living with partner	0.99 (0.96-1.02)	1.05 (1.01-1.10)
Separated, divorced or widowed	1.02 (0.98-1.05)	1.03 (0.97-1.09)
Employment Status (ref: Retired/not currently employed)		
Employed part-time (≤35 hours/week)	1.01 (0.98-1.04)	1.04 (0.99-1.09)
Employed full-time (>35 hours/week)	0.99 (0.97-1.02)	1.01 (0.97-1.05)
U.S.-born (ref: Not U.S.-born)	0.95 (0.91-0.99)	0.97 (0.92-1.02)

The table summarizes Poisson regression analyses that examined associations between participant characteristics and the perceived utility of genetic testing, rated on a 1-10 scale, with separate models for each gender. Estimates represent exponentiated coefficients, and show the relative increase in perceived efficacy scores relative to the reference category. Analyses included all covariates jointly, and were weighted to generate estimates valid for the HCHS/SOL target population. No findings were statistically significant at p<0.01.
