# Supplementary material for: Awareness and utilization of genetic testing among Hispanic and Latino adults living in the US: The Hispanic Community Health Study/Study of Latinos
Source: HGG Adv. 2022 Nov 19;4(1):100160. doi: 10.1016/j.xhgg.2022.100160 (PMC9722474; doi:10.1016/j.xhgg.2022.100160)
Supplement: Document S2. Article plus supplemental information [file mmc2.pdf]

HGG Advances

ARTICLE

Awareness and utilization of genetic testing among Hispanic and Latino adults living in the US: The Hispanic Community Health Study/Study of Latinos

Kurt D. Christensen,[1,2,3] Mengran Zhang,[4] Lauren N. Galbraith,[1,5] Einat Granot-Hershkovitz,[4,6] Sarah C. Nelson,[7] Sara Gonzalez,[8] Maria Argos,[9] Krista M. Perreira,[10] Martha L. Daviglus,[11,12] Carmen R. Isasi,[8] Jianwen Cai,[13] Gregory A. Talavera,[14] Carrie L. Blout Zawatsky,[2,15,16,17] Robert C. Green,[2,6,15,17] Rosario Isasi,[18] Robert Kaplan,[8,19] and Tamar Sofer[6,20,21,*]

Summary

We investigated the awareness, perceived usefulness, and use of genetic testing among Hispanic and Latino individuals. Annual follow-up surveys for the Hispanic Community Health Study/Study of Latinos (HCHS/SOL) from 2019 to April 2020 assessed participants' level of awareness and use of genetic tests to determine disease risks, likelihood of passing disease to children, disease treatment, or drug selection. They also were asked to rate the usefulness of the tests for managing a person's health on a 1 (not at all useful) to 10 (extremely useful) scale. There were 5,769 HCHS/SOL participants who completed at least one survey question. Of the target population, 55.2% was aware of at least one type of genetic test. Awareness varied between HCHS/SOL enrollment sites and was higher among individuals who had higher educational attainment and had higher incomes. Only 3.3% of the target population reported receiving one or more of the tests described. HCHS/SOL individuals rated the usefulness as 8.4, on average, with lower scores observed among U.S.-born individuals compared to individuals born outside the United States, with differences by HCHS/SOL enrollment sites. In conclusion, while awareness of genetic testing among Hispanic and Latino individuals varies by location, education, and income, perceptions about its usefulness are high while experiences with testing are rare. Results identify groups and locations that may benefit from greater outreach about the capabilities of genetic testing and precision medicine.

Introduction

The roles of genomic testing in all aspects of medicine are expanding rapidly. Already, genomic testing is accelerating diagnoses and identifying individuals with genetic predispositions for highly actionable conditions.[1–4] Pharmacogenomic (PGx) testing allows healthcare providers to tailor treatment decisions and medication dosing according to individuals' genomic profiles.[5–7] Preconception and prenatal genomic testing assists potential parents with reproductive decisions by informing them about their carrier status for genetic disorders.[8–10] These examples, among others, demonstrate the increasing usefulness and breadth of genomic testing applications in healthcare.

Analyses of genomic testing show uneven uptake between racial and ethnic groups, including Hispanic and Latino populations.[11–13] Many factors contribute to these disparities, including access to services, how often healthcare providers recommend testing, and logistical barriers. Awareness about genomic tests may be particularly important. Racial and ethnic minority populations typically report lower awareness and knowledge about genetic testing.[11,12] Hispanic and Latino populations merit special attention. Comprising more than 18% of the population, Hispanic and Latino populations are the largest racial and ethnic minority group in the United States. Numerous studies report that awareness and use of genetic testing among U.S. Hispanics and Latinos is low compared with non-Hispanics/Latinos.[14–21] In contrast, a survey conducted in 2017 showed no statistical differences by race and ethnicity in terms of knowledge about genetic testing. Studies that address characteristics associated with genetic

[1]PRecisiOn Medicine Translational Research (PROMoTeR) Center, Department of Population Medicine, Harvard Pilgrim Health Care Institute, Boston, MA, USA; [2]Broad Institute of Harvard and MIT, Cambridge, MA, USA; [3]Department of Population Medicine, Harvard Medical School, Boston, MA, USA; [4]Division of Sleep and Circadian Disorders, Brigham and Women's Hospital, Boston, MA 02115, USA; [5]Departments of Epidemiology and Biostatistics, Boston University of Public Health, Boston, MA, USA; [6]Department of Medicine, Harvard Medical School, Boston, MA, USA; [7]Department of Biostatistics, University of Washington, Seattle, WA, USA; [8]Department of Epidemiology and Population Health, Albert Einstein College of Medicine, Bronx, NY, USA; [9]School of Public Health, University of Illinois at Chicago, Chicago, IL, USA; [10]Department of Social Medicine, University of North Carolina, School of Medicine, Chapel Hill, NC, USA; [11]Institute for Minority Health Research, University of Illinois at Chicago, Chicago, IL, USA; [12]Feinberg School of Medicine, Northwestern University, Chicago, IL, USA; [13]Collaborative Studies Coordinating Center, Department of Biostatistics, University of North Carolina at Chapel Hill, Chapel Hill, NC, USA; [14]South Bay Latino Research Center, Department of Psychology, San Diego State University, San Diego, CA, USA; [15]Division of Genetics, Department of Medicine, Brigham and Women's Hospital, Boston, MA, USA; [16]MGH Institute of Health Professions, Boston, MA, USA; [17]Ariadne Labs, Boston, MA, USA; [18]Department of Human Genetics, University of Miami Miller School of Medicine, Miami, FL, USA; [19]Public Health Sciences Division, Fred Hutchinson Cancer Research Center, Seattle, WA, USA; [20]Division of Sleep and Circadian Disorders, Department of Medicine, Brigham and Women's Hospital, 221 Longwood Avenue, Suite 225C, Boston, MA 02115, USA
[21]Lead contact
*Correspondence: tsofer@bwh.harvard.edu
https://doi.org/10.1016/j.xhgg.2022.100160.
© 2022 The Author(s). This is an open access article under the CC BY license (http://creativecommons.org/licenses/by/4.0/).

testing awareness among Hispanic and Latino individuals are lacking.

Attitudes toward genomic testing, including perceived usefulness, may also influence the use of genetic tests. Perceived usefulness is a key component of numerous health behavior theories, including the health belief model, protection motivation theory, and adaptations of self-regulation theory to address genetic service use[22–24] and has consistently been shown to influence genomic testing uptake.[25,26] A number of studies suggest that, while most communities have positive attitudes toward genomic testing,[15] racial and ethnic minority communities tend to perceive less usefulness to genomic testing than non-Hispanic White populations. Limited research has examined factors that may influence the perceived usefulness of genomic testing specifically among Hispanics and Latinos.

We addressed this lack of data by surveying participants of the Hispanic Community Health Study/Study of Latinos (HCHS/SOL).[27] We expanded the annual follow-up survey administered by this large multi-center epidemiologic study in Hispanic and Latino populations to gather data about participants' awareness and use of different genetic tests. The goal of our study was to provide population-based insights about factors that may influence awareness, perceived usefulness, and genomic testing uptake.

Materials and methods

The HCHS/SOL

The HCHS/SOL is a population-based longitudinal cohort study established to study risk and protective factors in cardiovascular disease development among Hispanic and Latino individuals in the United States. Details of the HCHS/SOL study design and cohort have been reported previously.[27] Briefly, the study follows 16,415 Hispanic and Latino participants aged 18–74 years recruited in field centers from four metropolitan areas: The Bronx, New York; Miami, Florida; Chicago, Illinois; and San Diego, California. Individuals were recruited via probability sampling from pre-defined census block units chosen to provide diversity with respect to socioeconomic status as well as national origin or Hispanic or Latino background.[28] To recruit Spanish- or English-speaking individuals into the HCHS/SOL who self-identify as having a Hispanic or Latino background, research staff asked potential participants the following question during screening: "Do you consider yourself to be Hispanic or Latino?" If the question was not clear to the potential participant, the interviewer clarified with the statement, "We consider Hispanic/Latino individuals to be people from Latin America, South American, Central American, and the Caribbean." A baseline clinic visit took place from 2008 to 2011, and individuals participated in a second clinic visit 6 years later, on average. Participants were asked during their interview to self-identify with specific Hispanic or Latino background, "Which of the following best describes your Hispanic/Latino heritage?", with potential responses being Dominican or Dominican descent, Central American or Central American descent, Cuban or Cuban descent, Mexican or Mexican descent, Puerto Rican or Puerto Rican descent, South American or South American descent, more than one heritage, or other. Thus, individuals are identified with the following Hispanic or Latino backgrounds: Mexican, Central American, Cuban, Dominican, Puerto-Rican, South American, and other or multiple backgrounds. All study participants provided written informed consent at their recruitment center during their clinical exam. The informed consent form included the statement "I (agree/do not agree) to allow HCHS/SOL staff to contact me once a year to ask questions about my health and where I live." All enrolled individuals agreed to this condition and were asked to participate in a series of annual follow-up (AFU) phone calls. Protocols were approved by the institutional review boards at each institution of enrollment.

Measures of genetic testing awareness, use, and perceived benefits

Between April 2019 and April 2020, the HCHS/SOL AFU survey included items about genetic testing awareness, use, and perceived benefits (Figures S1 and S2). Awareness questions were adapted from items in the 2017 National Cancer Institute's Health Information National Trends Survey (HINTS),[29] asking respondents, "Have you heard of a genetic test to determine" each of the following: (1) "the risk or likelihood of getting a particular disease," (2) "the likelihood of passing an inherited disease to your children," (3) "to determine how a disease should be treated after diagnosis," and (4) "to determine which drug(s) may or may not work for an individual." Survey administrators classified responses as yes, no, or refuse to answer, with instructions to classify don't know and not sure responses as refusals. Don't know/not sure was omitted as a formal response option to mirror the design of the HINTS survey and facilitate data comparisons. All individuals who answered an item were asked, "If offered to you, would you be interested in receiving this kind of test?" with responses classified as yes, no, not sure/it depends, and refuse to answer. Individuals who responded yes about hearing about a type of genetic test were also asked if they had ever been offered such a test, and, if so, whether they had received the test. Response options on these types of questions were classified as yes, no, don't know, and refuse to answer. All survey participants were also asked to rate the usefulness they perceived in genetic testing. The question was presented as, "On a scale of 1–10, where 1 is 'not at all' and 10 is 'extremely,' how useful do you think genetic testing is for managing a person's health?", with response options ranging from 1 (not at all useful) to 10 (extremely useful). This item was adapted from a pilot clinical trial of genomic sequencing.[30]

The survey was administered in either English or Spanish, depending on participant preference. Survey items are presented in the Appendix.

Covariates

We extracted fixed and time varying covariates from the data collected at the most recent HCHS/SOL examination available for each individual. Covariates included age at the time of survey completion (categorized as 25–40, 41–60, or ≥61 years), gender, study center (Bronx, Chicago, Miami, or San Diego), self-reported Hispanic or Latino background (Dominican, Mexican, Central American, Cuban, Puerto Rican, South American, or more than one/other), educational attainment (less than high school degree, high school degree, associate, bachelor, or vocational degree, or masters, doctoral or professional degree), household income level (<\$10,000, \$10,001–20,000, \$20,001–40,000, \$40,001–75,000, or >\$75,000), current health insurance status, number of doctor visits within the prior year at the baseline visit (categorized to no visits, 1–2 visits, or ≥3), employment status (retired/not

currently employed, not retired but not currently employed, employed part time [≤35 h a week], or employed full time [>35 h a week]), nativity (born in the 50 U.S. states or not), and marital status (single, married or living with a partner, separated, divorced or widowed). Marital status was available only from the baseline exam and second AFU year (7–9 years before the genetic testing survey items were administered). Although a question about self-identified race was administered to HCHS/SOL participants, it was not used in the analyses because the majority of HCHS/SOL participants refused to answer the question or reported other. In computation of the inverse probability weights (described below) we also used sampling strata as a covariate.

Computation of inverse probability weights for population-representative estimates

To obtain estimates that were applicable to the target population represented by the HCHS/SOL, we applied inverse probability weighting (IPW) to respondents' data. Weights were estimated as $\frac{1}{P_{gts|AFU}} \times \frac{1}{P_{AFU|sol}} \times \frac{1}{P_{sol}}$, where P_{sol} was the probability of a person from the target population participating in HCHS/SOL, $P_{AFU|sol}$ was the probability of an HCHS/SOL subject participating in the relevant AFU survey, and $P_{gts|AFU}$ was the probability of AFU survey participants responding with a yes or no response to at least one of the genetic testing awareness items (e.g., if an individual was classified as refuse to answer to all four testing awareness questions, they were considered as non-respondent). We estimated $P_{AFU|sol}$ and $P_{gts|AFU}$ using logistic regression. Logistic regression used all covariates described above. For time-varying covariates, we used the measured values from visit 1 to compute $P_{AFU|sol}$, and, when possible, values from visit 2 to compute $P_{gts|AFU}$. Missing data in these models were imputed via fully conditional specification using the *mice* R package (version 3.13.0) with five iterations to create each of five imputed datasets. For each imputed dataset, we obtained estimates of $logit(P_{AFU|sol}) = x^T\widehat{\beta}_{AFU}$ and $logit(P_{gts|AFU}) = x^T\widehat{\beta}_{gts}$, and then averaged them. The final estimates $\widehat{P}_{AFU|sol}$ and $\widehat{P}_{gts|AFU}$ are based on these averages, e.g., $\widehat{P}_{AFU|sol} = logit^{-1}(x^T\widehat{\beta}_{AFU})$ where $logit^{-1}(\cdot)$ is the inverse of the logistic function and $x^T\widehat{\beta}_{AFU}$ is the average of the estimates across the five imputed datasets. While we used imputation to generate IPW weights, in association analyses when adjusting to covariates, we performed a complete case analysis. In secondary analyses, we also computed the IPW for participation in the AFU as $\frac{1}{P_{AFU|sol}} \times \frac{1}{P_{sol}}$.

Data analyses

All analyses, including computations of percentages and association analyses, accounted for the stratified sampling strategy and clustering of HCHS/SOL participants by applying functions from the *survey* R package (version 4.0).

Primary analyses of genetic test awareness used a composite measure, where individuals were assigned value of 1 (aware) if they reported awareness of any of the four types of genetic tests described and 0 (unaware) if they were not aware of any test and answered at least one item about genetic test awareness. Individuals with no data on any genetic test awareness items because of refusals and missing data were considered non-respondent and omitted from analyses (although they were taken into account in the IPW computation). A similar approach was used to classify whether participants were interested in these genetic tests, as well as to classify whether they had ever been offered or used them (individuals who were unaware of these tests were classified as never being offered them, and individuals who were never offered these tests were classified as never using them, in agreement with the survey's skip pattern). Participants who were classified as don't know/not sure on items about being offered or using genetic were included in analyses as not being offered or using those tests.

We identified demographic and healthcare-related factors associated with genetic testing awareness using logistic regression. We identified demographic and healthcare-related factors associated with perceived usefulness using Poisson regression, because of the highly skewed distribution of the responses. We used contrasts to compare awareness and perceived usefulness of genetic tests between extreme levels of variables associated with awareness and perceived usefulness.

Because of the small numbers of participants reporting being offered or using genetic tests, we performed unadjusted analyses reporting overall numbers of individuals and percentages for these outcomes, without performing association analysis. Findings were reported as statistically significant if p values were less than 0.01.

In secondary analyses, we compared the characteristics of participants who participated in the AFU but did not respond to the genetic testing survey to those who responded (unadjusted analysis), and repeated (fully adjusted) regression analyses of genetic testing awareness and perceived usefulness stratified by gender and by HCHS/SOL study center. Also, in supplemental analyses, we repeated the main analyses stratified by study center, because there could be systematic differences between regions with respect to genetic testing.

Results

Of 16,415 HCHS/SOL visit 1 participants, 9,408 participated in the study's 2019–2020 AFU phone survey and 5,769 completed at least one item about genetic testing for an overall participation rate of 35.1% (unweighted), or 32.6% of the target population (weighted estimate). Individuals who participated in the AFU but did not complete any genetic testing awareness items tended to be less educated, had lower household incomes, were more likely to be single and retired, and were more likely to be enrolled at the Bronx site and less likely to be enrolled at the Chicago and San Diego sites than individuals who completed at least one awareness item (all $p < 0.001$) (Table S1). Characteristics of HCHS/SOL participants who were analyzed and the target population their represent are summarized in Table 1. Visualization of missingness patterns in the dataset across all HCHS/SOL individuals, participants in the AFU, and genetic testing questionnaire responders are provided in the dedicated GitHub repository. Overall, the highest missingness was in the income level variable, followed by reported doctor visits.

Overall, 2,891 survey responders, or 55.3% (weighted percentage) of individuals in the target population reported awareness of at least one of the four types of genetic tests. These included tests to determine risks for particular diseases, tests to determine the likelihood of passing inherited risks to children, tests to inform disease treatment, and tests to determine medication selection. Individuals were most likely to report awareness of genetic tests to determine risks of getting disease (2,089 participants,

Table 1. Demographic characteristics, reported as unweighted n and weighted %, representative of the HCHS/SOL target population

		Site				
	Overall	Bronx	Chicago	Miami	San Diego	% Missing
Total N	5,769	1.030	1.950	1.065	1.724	
Age, years						0
25–40	1,000 (30.7)	198 (33.4)	377 (37.6)	137 (21.6)	288 (32.9)	
41–60	2,674 (43.1)	442 (40.6)	916 (42.7)	508 (45.5)	808 (43.6)	
61 or older	2,095 (26.2)	390 (26.0)	657 (19.7)	420 (32.9)	628 (23.5)	
Gender						0
Female	3,701 (52.4)	679 (54.5)	1,163 (48.1)	713 (52.9)	1,146 (52.1)	
Male	2,068 (47.6)	351 (45.5)	787 (51.9)	352 (47.1)	578 (47.9)	
Education						0.4
Less than high school degree	2,004 (31.6)	381 (39.8)	894 (42.9)	203 (20.6)	526 (27.1)	
High school degree	1,458 (27.4)	258 (25.6)	494 (30.0)	282 (28.4)	424 (26.8)	
Associate, bachelor, or vocational degree	2,078 (37.5)	345 (31.8)	516 (25.1)	521 (46.1)	696 (42.2)	
Masters, doctoral, professional degree	206 (3.5)	41 (2.8)	38 (1.9)	57 (4.8)	70 (3.9)	
Household income						2.5
Less than $10,000	613 (10.9)	188 (16.5)	150 (6.8)	135 (11.4)	140 (6.9)	
$10,001–$20,000	1,532 (27.3)	316 (33.4)	497 (24.1)	334 (30.0)	385 (19.6)	
$20,001–$40,000	1,999 (33.7)	261 (28.3)	757 (38.6)	360 (36.1)	621 (34.5)	
$40,001–$75,000	1,039 (18.6)	146 (15.0)	369 (22.3)	150 (15.6)	374 (23.4)	
More than $75,000	441 (9.6)	81 (6.9)	130 (8.2)	56 (6.9)	174 (15.7)	
Health insurance status						0.3
Uninsured	1,686 (31.6)	121 (17.4)	738 (43.9)	395 (38.5)	432 (32.4)	
Has health insurance	4,068 (68.4)	899 (82.6)	1,212 (56.1)	669 (61.5)	1,288 (67.6)	
Doctor visit in last 12 months						1.3
No	1,521 (32.2)	133 (20.0)	531 (33.4)	441 (44.6)	416 (31.2)	
Yes, one or two times	1,820 (29.9)	309 (32.4)	635 (31.4)	276 (22.8)	600 (33.7)	
Yes, at least three times	2,352 (38.0)	544 (47.6)	778 (35.3)	332 (32.7)	698 (35.1)	
Hispanic or Latino background						0.3
Dominican	413 (9.8)	380 (31.9)	15 (0.5)	17 (1.3)	1 (0.1)	
Mexican	2,753 (38.3)	46 (10.3)	1,093 (62.3)	8 (0.9)	1,606 (92.7)	
Central American	575 (6.9)	56 (4.2)	226 (7.2)	271 (15.2)	22 (0.8)	
Cuban	593 (19.4)	8 (1.2)	17 (1.5)	565 (67.8)	3 (0.4)	
Puerto Rican	820 (16.8)	442 (43.7)	342 (18.5)	19 (2.3)	17 (1.5)	
South American	424 (5.1)	44 (3.9)	201 (6.9)	157 (9.5)	22 (0.8)	
More than one/other	172 (3.7)	48 (4.8)	53 (3.1)	26 (2.9)	45 (3.6)	
Language preference						0
Spanish	4,731 (74.7)	679 (58.8)	1,676 (79.8)	1,030 (94.3)	1,346 (68.8)	
English	1,038 (25.3)	351 (41.2)	274 (20.2)	35 (5.7)	378 (31.2)	
Marital status						0.1
Single	1,234 (30.3)	363 (42.4)	336 (25.1)	234 (26.6)	301 (23.8)	
Married or living with partner	3,288 (52.3)	421 (40.6)	1,265 (61.7)	532 (49.7)	1,070 (62.0)	

(*Continued on next page*)

Table 1. *Continued*

	Overall	Site: Bronx	Chicago	Miami	San Diego	% Missing
Total N	5,769	1.030	1.950	1.065	1.724	
Separated, divorced or widowed	1,240 (17.4)	244 (17.1)	349 (13.2)	298 (23.7)	349 (14.2)	
Employment status						0.3
Retired/not currently employed	2,362 (39.3)	507 (46.8)	714 (30.3)	427 (40.6)	714 (36.0)	
Employed part time (≤35 h/week)	1,318 (21.2)	210 (18.9)	443 (22.8)	251 (20.8)	414 (22.8)	
Employed full time (>35 h/week)	2,071 (39.5)	298 (34.3)	793 (46.9)	387 (38.6)	593 (41.3)	
Nativity						0.3
Not U.S. born	4,835 (77.2)	786 (70.1)	1,684 (77.7)	,1017 (92.2)	1,348 (69.5)	
U.S. born	918 (22.8)	239 (29.9)	263 (22.3)	46 (7.8)	370 (30.5)	

target population weighted percentage 40.3%) and the likelihood of passing disease to children (2,059 participants, weighted percentage 42.1%), and least likely to know about genetic tests providing information about how diseases should be treated (874 participants, weighted percentage 16.6%) or determining medication selection (885 individuals, weighted percentage 16.4%). Few individuals reported ever being offered or receiving the types of genetic tests described in the survey (Figure 1). Only 362 respondents (weighted percentage 6.5%) reported being offered any of the four types of tests described, and 190 respondents (weighted percentage 3.3%) reported receiving at least one of the tests described. When restricted to individuals who reported an awareness of at least one type of genetic test, analyses showed that 11.7% of the target population (362 respondents) reported being offered at least one of the tests described, while 6.0% of the target population (190 respondents) reported receiving at least one of the tests (Figure S3). Findings were consistent in analyses that were stratified by gender (Figure S4).

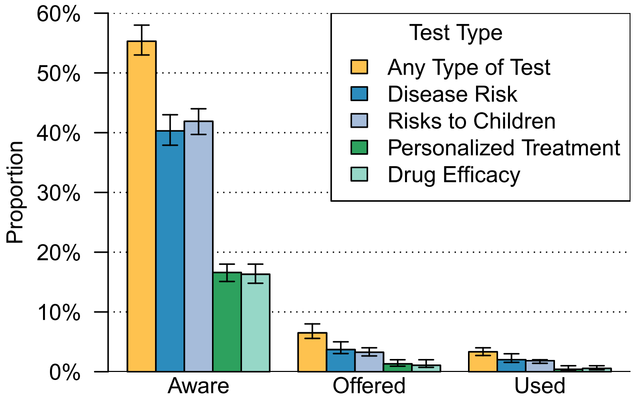

Figure 1. Percentages of the HCHS/SOL target population who report awareness of specific types of genetic tests. Proportions and 95% confidence intervals, represented as bars in the figure, were computed in a weighted analysis to be representative of the HCHS/SOL target population. Proportions were multiplied by 100 to obtain percentages.

Few demographic or medical care factors that were examined by logistic regression analyses were associated with overall awareness of genetic testing (Table 2). Some of the greatest differences were observed between HCHS/SOL field centers (Figure S5). Awareness was greatest among individuals enrolled at the Miami site (weighted percentage 67.2%), and lowest among individuals enrolled at the Chicago site (weighted percentage 36.2%) (odds ratio [OR], 3.39; 95% confidence interval [CI], 2.44–4.71, $p < 0.001$). Income was also associated with genetic testing awareness: 73.3% (weighted percentage) of individuals with household incomes of more than \$75,000 reported awareness, compared with 46.3% (weighted percentage) with incomes of less than \$10,000 (OR, 2.53; 95% CI, 1.60–3.99; $p < 0.001$). Finally, we observed differences by educational attainment: 66.2% of individuals with associate, bachelor, or vocational degree reported awareness, compared with 45.0% of individuals with less than a high school degree (OR, 1.65; 95% CI, 1.33–2.05; $p < 0.001$).

Stratified analyses showed that some of the above-mentioned patterns were stronger at specific sites (Table S2). Differences by educational attainment only emerged for individuals enrolled at the Chicago site, while differences by income only for individuals enrolled at the San Diego site. Differences by gender were not observed overall, and gender-specific analyses (Table S3) generally showed similar differences by educational attainment. Differences by site were driven mainly by men, while differences by income were driven mainly by women.

Analyses about specific genetic tests showed similar trends (Table S4). Differences by education were observed on each of the items asking about awareness of specific tests (OR between more than high school degree vs less than high school degree ≥1.70; $p < 0.001$ for all tests). Differences by income were observed for knowledge of tests for disease risk and risks to children (OR between >\$75,000 vs <\$10,000 ≥ 1.84; $p < 0.01$ for both tests). Differences in awareness by study site were observed only in analyses of genetic testing for risks of getting disease (OR of individuals at Miami vs Bronx site, 3.02; 95% CI, 2.11–4.31; $p < 0.001$). Finally, 11.6% of

Table 2. Summary of logistic regression models that examine associations between participant characteristics and awareness of at least one of the four types of genetic testing (n = 5,768)

Term	N aware in stratum	Weighted proportion	OR (95% CI)
Age, years (ref: 40 and younger)	538	0.58	
41–60	1,355	0.55	1.02 (0.82–1.27)
61 or older	998	0.52	0.95 (0.70–1.28)
Gender (ref: female)	1,901	0.56	
Male	990	0.55	0.88 (0.73–1.06)
Education, years (ref: less than high school degree)	798	0.45	
High school degree	690	0.51	1.04 (0.83–1.30)
Associate, bachelor, or vocational degree	1,258	0.66	1.65 (1.33–2.05)**
Masters, doctoral, professional degree	137	0.65	1.48 (0.88–2.50)
Center (ref: Bronx)	540	0.52	
Chicago	697	0.36	0.54 (0.39–0.75)**
Miami	692	0.67	1.80 (1.25–2.60)*
San Diego	962	0.58	1.17 (0.78–1.76)
Income (ref: Less than $10,000)	282	0.46	
$10,001–$20,000	711	0.51	1.14 (0.84–1.55)
$20,001–$40,000	986	0.53	1.24 (0.91–1.69)
$40,001–$75,000	558	0.62	1.77 (1.24–2.52)*
More than $75,000	290	0.73	2.53 (1.60–3.99)**
Health Insurance Status (ref: uninsured)	790	0.53	
Has health insurance	2,093	0.56	1.02 (0.81–1.27)
Doctor Visit in Last 12 Months (ref: none)	762	0.56	
Yes, one or two times	925	0.55	0.98 (0.77–1.24)
At least three times	1,165	0.55	1.00 (0.77–1.29)
Hispanic or Latino Background (ref: Dominican)	222	0.51	
Mexican	300	0.57	0.98 (0.63–1.54)
Central American	392	0.68	1.07 (0.67–1.73)
Cuban	1,269	0.51	1.21 (0.74–1.97)
Puerto Rican	381	0.53	0.98 (0.67–1.43)
South American	207	0.71	0.95 (0.60–1.51)
More than one/other	112	0.51	1.47 (0.80–2.72)
Language Preference (ref: Spanish)	2,273	0.53	
English	618	0.62	1.18 (0.91–1.54)
Marital Status (ref: Single)	673	0.6	
Married or living with partner	1,627	0.55	0.84 (0.68–1.05)
Separated, divorced or widowed	586	0.49	0.69 (0.52–0.93)
Employment Status (ref: Retired/not currently employed)	1,128	0.53	
Employed part time (≤35 h/week)	694	0.57	1.08 (0.84–1.39)
Employed full time (>35 h/week)	1,058	0.57	0.97 (0.77–1.21)

(*Continued on next page*)

Table 2. *Continued*

Term	N aware in stratum	Weighted proportion	OR (95% CI)
Nativity (ref: Not U.S. born)	2,339	0.53	
U.S. born	545	0.62	1.14 (0.86–1.52)

Analyses included all covariates jointly, and were weighted to generate estimates representative of the HCHS/SOL target population. N aware in stratum further provide the raw number of individuals aware of at least one type of test, and their proportion in the strata, weighted to represent the HCHS/SOL target population, is provide in the column weighted proportion.
*$p < 0.01$.
**$p < 0.001$.

individuals who were separated, divorced, or widowed reported awareness of drug efficacy genetic tests, compared to 17.5% of individuals who were married or living with a partner (OR, 0.66; 95% CI, 0.48–0.89; $p = 0.007$). We did not run models to examine factors associated with being offered or using specific tests because the numbers of participants were small, but unadjusted, weighted percentages are summarized in Figure 1.

Overall, participants rated the usefulness of genetic testing for managing a person's health 8.4 (standard error = 0.05), on average, on a 1 (not at all useful) to 10 (extremely useful) scale. Few demographic factors were associated with usefulness scores in Poisson regression models (Table 3). Analyses showed that individuals enrolled at the Miami site rated the usefulness of genetic testing 7% higher than participants enrolled at the Bronx site, on average (95% CI, 2%–12% higher; $p = 0.004$). Conversely, mean perceived usefulness scores were 6% lower among U.S.-born individuals than individuals born outside the United States (95% CI, 4%–9% lower; $p < 0.001$). Site-specific analyses (Table S5) were generally consistent with these findings, although some differences by Hispanic or Latino background were observed. No demographic factors were associated with perceived usefulness in gender-specific analyses (Table S6).

Discussion

We leveraged one of the largest, population-based cohort studies of U.S. Hispanic and Latino individuals to provide novel insights about demographic factors associated with genetic testing awareness in this growing population. Our findings showed that approximately 55% of this population was aware of genetic tests for health. This proportion was only slightly higher than findings from the 2017 HINTS, where just under one-half of Hispanic and Latino respondents and 57% of respondents overall reported awareness of health-related genetic testing.[31] Similar to prior work that included all population groups, awareness of PGx applications, including tailored treatment, was much lower than awareness about personal disease risks or risks to children in our population.[31] The rates that individuals reported being offered genetic testing were low, and only 3% reported ever using these tests.

Our findings highlight concerns about the potential for important segments of Hispanic and Latino populations to miss opportunities to benefit from improvements in genetic testing. Tremendous advances have occurred in medical genetics in recent years. These include the refinement of approaches to assess genetic risk, such as polygenic risk predictions,[32,33] evolutions in the use of sequencing for expanded carrier screening,[8,9] successes in tumor sequencing to guide cancer treatments, and continued development of best practices for PGx applications.[6,34] The potential for genomics to revolutionize population health will not be realized without substantial efforts to improve how often Hispanic and Latino individuals are offered genetic services and facilitating access to testing and counseling.[35,36] HCHS/SOL's enrollment of more than 16,000 Hispanic and Latino individuals to date provides an instructive model for other cohort studies to consider to address awareness limitations in populations that are traditionally under-represented in biomedical research. Our study found that few Hispanic and Latino participants had even been offered services, a finding that has been observed in clinical settings.[13] Efforts need to be improved to ensure Hispanic and Latino individuals who meet the criteria for a genetics referral are informed of such services and their potential benefits. Such efforts may need to be complemented with policies that address access barriers if we hope to achieve improvements in clinical settings.

A number of differences observed in prior work were not observed in our analyses. Specifically, differences in Hispanic and Latino populations by gender, Hispanic or Latino background, and birth location reported from the 2005 HINTS data were not statistically significant in our analyses. Only a few demographic factors were associated with genetic testing awareness in our study population. Specifically, we observed differences in awareness between centers of enrollment and by income and education. A greater awareness of genetic testing among individuals with higher educational status and income was consistent with previous findings.[37,38] Geographic differences were unexpected and large. We found the least awareness among Hispanic and Latino individuals at the Chicago site and the greatest awareness among Hispanic and Latino individuals at Miami, findings that that persisted after adjustments for demographic characteristics such as socioeconomic status and language preferences. In contrast, prior reports of HINTS data had shown the greatest awareness in 2005 among respondents from the Midwest and

Table 3. Summary of a Poisson regression model that examines associations between participant characteristics and the perceived usefulness of genetic testing, rated on a 1 (not at all useful) to 10 (extremely useful) scale

Term	Estimate (95% CI)
Age, years (ref: 40 and younger)	
41–60	1.03 (1.00–1.06)
61 or older	0.98 (0.95–1.02)
Male gender (ref: female)	0.98 (0.96–1.00)
Education, years (ref: less than high school degree)	
High school degree	1.01 (0.98–1.04)
Associate, bachelor, or vocational degree	1.03 (1.00–1.06)
Masters, doctoral, professional degree	0.97 (0.92–1.02)
Center (ref: Bronx)	
Chicago	1.01 (0.96–1.06)
Miami	1.07 (1.02–1.12)*
San Diego	1.03 (0.97–1.09)
Income (ref: Less than $10,000)	
$10,001–$20,000	1.01 (0.96–1.06)
$20,001–$40,000	1.01 (0.96–1.06)
$40,001–$75,000	1.03 (0.98–1.08)
More than $75,000	0.98 (0.93–1.04)
Has health insurance status (ref: uninsured)	1.00 (0.98–1.02)
Doctor visit (ref: none)	
Yes, one or two times	1.00 (0.98–1.03)
More than three times	1.01 (0.98–1.03)
Hispanic or Latino background (ref: Dominican)	
Mexican	0.99 (0.93–1.06)
Central American	1.00 (0.95–1.05)
Cuban	1.03 (0.97–1.09)
Puerto Rican	1.00 (0.95–1.05)
South American	0.99 (0.94–1.05)
More than one/other	1.00 (0.94–1.07)
English language preference (ref: Spanish)	0.95 (0.92–0.98)*
Marital status (ref: single)	
Separated, divorced or widowed	1.02 (0.98–1.05)
Married or living with partner	1.02 (0.99–1.06)
Employment status (ref: retired/not currently employed)	
Employed part time (≤35 h/week)	1.02 (1.00–1.05)
Employed full time (>35 h/week)	1.00 (0.97–1.02)
U.S. born (ref: Not U.S. born)	0.96 (0.93–0.99)*

Estimates represent exponentiated coefficients, and show the relative increase in perceived efficacy scores relative to the reference category. Analyses included all covariates jointly, and were weighted to generate estimates valid for the HCHS/SOL target population.
*$p < 0.01$.
**$p < 0.001$.

the lowest awareness among respondents from the South, although regional differences in HINTS data were not observed in 2010 data.[37,39] It is possible that the differences we observed reflect current differences by location, such as differences in the way genetic testing services are advertised by city and target audience or the way genetic

testing is regulated. Lower awareness about direct-to-consumer tests have also been observed previously among individuals living in rural areas compared with urban areas,[40] suggesting that the geographic breadth of enrollment at particular HCHS/SOL centers may have also affected awareness. Regardless, the large variation by geography likely reflects the wide diversity of experiences and attitudes within the Hispanic and Latino community.[41,42] Education and outreach efforts that are targeted to the beliefs, needs, and concerns of Hispanic and Latino residents of specific communities are likely to yield the greatest improvements in awareness of genetic services.

Our study also showed that very few Hispanic and Latino participants remembered being offered or receiving genetic testing. HINTS data across populations from 2017 showed that 12% of respondents overall reporting ever having undergone a genetic test.[31] In contrast, only 3% of respondents in our study reported ever receiving a genetic test. Differences in survey administration may explain some of these differences, as the 2017 HINTS instrument asked about a more expansive set of tests, including ancestry testing, paternity testing, and DNA fingerprinting.[31] Additional work may be beneficial to provide insight about potential disparities by ethnicity in genetic testing use.

Our work also showed relatively high perceptions among Hispanic and Latino participants about the usefulness of genetic testing. Interestingly, we observed greater perceived usefulness of genetic testing among individuals who were not U.S. born, which is contrasts with prior work with Hispanic and Latinos populations, which has found a lower awareness among individuals of lower acculturation and those who have resided in the United States for shorter times.[39,43] Studies have also found that Hispanic and Latino individuals with lower acculturation cite more barriers and perceived harms from genetic testing as well. Moreover, in previous findings from HCHS/SOL, we found that individuals with lower acculturation (not born within the 50 U.S. states, prefer Spanish over English) refused to share their genetic data with for-profit organizations at higher rates compared with those with higher acculturation.[42] Our findings also highlight challenges to addressing existing disparities in the epidemiological evidence about genomics[44–48] and efforts to enrich the participation of Hispanic and Latino individuals in precision medicine initiatives such as the All of Us Research Program.[38,49] It is possible that attitudes about genetic testing differ in the countries of birth of our survey respondents; more positive attitudes and greater rates of perceived usefulness have been reported in studies conducted outside the United States, although such studies were not conducted in countries with primarily Hispanic and Latino populations.[50–52]

The strengths of our study include the large sample size of an understudied population and post hoc adjustment to provide population-based estimates. Our analytic sample not only focused on Hispanics and Latino individuals, but also included large numbers of respondents with lower educational attainment and income. Limitations to our analyses included moderate completion rates for the genetic awareness questions and the use of brief questions that provide limited insight about the barriers to awareness and test use or why participants felt genetic testing was useful or not useful. We did not study psychosocial and cultural factors, such as religiosity, medical distrust, and acculturation, which may vary within the Hispanic and Latino population and may be associated with outcomes of interest, which remains a topic of future work. Participants were enrolled at four centers with limited representation from rural areas. Our descriptions of genetic tests were adapted from items used previously in the national HINTS instrument and do not reflect the range of health-related applications that currently exist. Furthermore, response rates to the HCHS/SOL AFU survey were moderate, and numerous individuals who started the survey did not provide data for the genetic testing section. Don't know responses were analyzed as no throughout, and the findings may underestimate the proportion of respondents with genetic test awareness, as well as the proportion of respondents who had been offered and used genetic tests.

Nevertheless, our work adds weight to evidence that use of genetic testing is limited in Hispanic and Latino individuals, although attitudes toward these applications are largely positive. These findings highlight the need to tailor genetic testing education and outreach to account for the wide variation in perception, needs, and potentially, believes, among Hispanic and Latino individuals. Education and outreach that accounts for the large diversity of Hispanic and Latino communities are likely to yield the greatest improvements in genetic test awareness and better ensure all individuals are able to capitalize on improvements in genomic medicine.

Data availability

HCHS/SOL data are available via a data use agreement with the HCHS/SOL Data Coordinating Center. See https://sites.cscc.unc.edu/hchs/ for study procedures. HCHS/SOL data are also available on the National Heart Lung and Blood Institute's BioLINCC (Biologic Specimen and Data Repository Information Coordinating Center) repository under accession number HLB01141422a.

Code availability

Code is publicly available on the GitHub repository: https://github.com/tamartsi/Genetic_testing_awareness_SOL.

Ethics declaration

The HCHS/SOL was approved by the institutional review boards at each field center, where all participants gave written informed consent during their clinical exam, and by the Non-Biomedical IRB at the University of North Carolina at Chapel Hill, to the HCHS/SOL Data

Coordinating Center. The informed consent form included the statement "I (agree/do not agree) to allow HCHS/SOL staff to contact me once a year to ask questions about my health and where I live."

Supplemental information

Supplemental information can be found online at https://doi.org/10.1016/j.xhgg.2022.100160.

Acknowledgments

The authors thank the staff and participants of HCHS/SOL for their important contributions. The investigators website is: http://www.cscc.unc.edu/hchs/. The authors acknowledge the valuable contributions of Dr. Gerardo Heiss, a pillar of the community, a PI of the HCHS/SOL, and a contributor to this study. Dr. Heiss passed away in June 2022. The Hispanic Community Health Study/Study of Latinos is a collaborative study supported by contracts from the National Heart, Lung, and Blood Institute (NHLBI) to the University of North Carolina (HHSN268201300001I/N01-HC-65233), University of Miami (HHSN268201300004I/N01-HC-65234), Albert Einstein College of Medicine (HHSN268201300002I/N01-HC-65235), University of Illinois at Chicago (HHSN268201300003I/N01-HC-65236 Northwestern University), and San Diego State University (HHSN268201300005I/N01-HC-65237). The following institutes, centers, and offices have contributed to the HCHS/SOL through a transfer of funds to the NHLBI: National Institute on Minority Health and Health Disparities, National Institute on Deafness and Other Communication Disorders, National Institute of Dental and Craniofacial Research, National Institute of Diabetes and Digestive and Kidney Diseases, National Institute of Neurological Disorders and Stroke, and the NIH Institution-Office of Dietary Supplements. K.D.C. was supported by NIH grant K01-HG009173.

Author contributions

Conceptualization: K.D.C., L.N.G., C.L.B.Z., and T.S.; data curation: M.Z. and T.S.; formal analysis: M.Z. and T.S.; funding acquisition: R.I., R.K., T.S. C.I., and M.L.D.; investigation: R.I. and R.K.; methodology: K.D.C., C.L.B.Z, R.I., R.K., and T.S.; project administration: resources: R.C.G., R.I., R.K., and T.S.; supervision: R.I., R.K., and T.S.; validation: T.S.; visualization: M.G. and T.S.; writing – original draft: K.D.C., L.N.G., M.Z., and T.S.; writing – review and editing: K.D.C., L.N.G., K.P., T.S., C.L.B.Z, M.A., C.I., M.L.D., G.A.T., and G.H.

Declaration of interests

K.D.C. was supported by a research grant from Sanford Health. R.C.G. has received compensation for advising the following companies: AIA, Embryome, Genome Web, Genomic Life, Grail, Humanity, OptumLabs, Plumcare, Verily; and is co-founder of Genome Medical, Inc. All other authors declare no competing interests.

Received: August 24, 2022
Accepted: November 16, 2022

Web resources

GitHub page with code and results used in this manuscript: https://github.com/tamartsi/Genetic_testing_awareness_SOL

Hispanic Community Health Study/Study of Latinos website: https://sites.cscc.unc.edu/hchs/

National Human Genome Research Institute. Genome Statute and Legislation Database: https://www.genome.gov/about-genomics/policy-issues/Genome-Statute-Legislation-Database

U.S. Census Bureau: QuickFacts. https://www.census.gov/quickfacts/fact/table/US

References

1. Esplin, E.D., Oei, L., and Snyder, M.P. (2014). Personalized sequencing and the future of medicine: discovery, diagnosis and defeat of disease. Pharmacogenomics *15*, 1771–1790.
2. Berg, J.S., Agrawal, P.B., Bailey, D.B., Jr., Beggs, A.H., Brenner, S.E., Brower, A.M., Cakici, J.A., Ceyhan-Birsoy, O., Chan, K., Chen, F., et al. (2017). Newborn sequencing in genomic medicine and public health. Pediatrics *139*, e20162252.
3. Amendola, L.M., Dorschner, M.O., Robertson, P.D., Salama, J.S., Hart, R., Shirts, B.H., Murray, M.L., Tokita, M.J., Gallego, C.J., Kim, D.S., et al. (2015). Actionable exomic incidental findings in 6503 participants: challenges of variant classification. Genome Res. *25*, 305–315.
4. Kalia, S.S., Adelman, K., Bale, S.J., Chung, W.K., Eng, C., Evans, J.P., Herman, G.E., Hufnagel, S.B., Klein, T.E., Korf, B.R., et al. (2017). Recommendations for reporting of secondary findings in clinical exome and genome sequencing, 2016 update (ACMG SF v2.0): a policy statement of the American College of Medical Genetics and Genomics. Genet. Med. *19*, 249–255.
5. O'Donnell, P.H., Wadhwa, N., Danahey, K., Borden, B.A., Lee, S.M., Hall, J.P., Klammer, C., Hussain, S., Siegler, M., Sorrentino, M.J., et al. (2017). Pharmacogenomics-based point-of-care clinical decision support significantly alters drug prescribing. Clin. Pharmacol. Ther. *102*, 859–869.
6. Roden, D.M., McLeod, H.L., Relling, M.V., Williams, M.S., Mensah, G.A., Peterson, J.F., and Van Driest, S.L. (2019). Lancet *394*, 521–532.
7. Relling, M.V., and Evans, W.E. (2015). Pharmacogenomics in the clinic. Nature *526*, 343–350.
8. Beauchamp, K.A., Muzzey, D., Wong, K.K., Hogan, G.J., Karimi, K., Candille, S.I., Mehta, N., Mar-Heyming, R., Kaseniit, K.E., Kang, H.P., et al. (2018). Systematic design and comparison of expanded carrier screening panels. Genet. Med. *20*, 55–63.
9. Kraft, S.A., Duenas, D., Wilfond, B.S., and Goddard, K.A.B. (2019). The evolving landscape of expanded carrier screening: challenges and opportunities. Genet. Med. *21*, 790–797.
10. Monaghan, K.G., Leach, N.T., Pekarek, D., Prasad, P., Rose, N.C.; and ACMG Professional Practice and Guidelines Committee (2020). The use of fetal exome sequencing in prenatal diagnosis: a points to consider document of the American College of Medical Genetics and Genomics (ACMG). Genet. Med. *22*, 675–680.
11. Suther, S., and Kiros, G.E. (2009). Barriers to the use of genetic testing: a study of racial and ethnic disparities. Genet. Med. *11*, 655–662.
12. Canedo, J.R., Miller, S.T., Myers, H.F., and Sanderson, M. (2019). Racial and ethnic differences in knowledge and attitudes about genetic testing in the US: systematic review. J. Genet. Couns. *28*, 587–601.

13. Cragun, D., Weidner, A., Kechik, J., and Pal, T. (2019). Genetic testing across young Hispanic and non-Hispanic white breast cancer survivors: facilitators, barriers, and awareness of the Genetic Information Nondiscrimination Act. Genet. Test. Mol. Biomarkers *23*, 75–83.
14. Agurs-Collins, T., Ferrer, R., Ottenbacher, A., Waters, E.A., O'Connell, M.E., and Hamilton, J.G. (2015). Public awareness of direct-to-consumer genetic tests: findings from the 2013 U.S. Health Information National Trends Survey. J. Cancer Educ. *30*, 799–807.
15. Hann, K.E.J., Freeman, M., Fraser, L., Waller, J., Sanderson, S.C., Rahman, B., Side, L., Gessler, S., Lanceley, A.; and PROMISE study team (2017). Awareness, knowledge, perceptions, and attitudes towards genetic testing for cancer risk among ethnic minority groups: a systematic review. BMC Publ. Health *17*, 503.
16. Cragun, D., Weidner, A., Lewis, C., Bonner, D., Kim, J., Vadaparampil, S.T., and Pal, T. (2017). Racial disparities in BRCA testing and cancer risk management across a population-based sample of young breast cancer survivors. Cancer *123*, 2497–2505.
17. Shields, A.E., Burke, W., and Levy, D.E. (2008). Differential use of available genetic tests among primary care physicians in the United States: results of a national survey. Genet. Med. *10*, 404–414.
18. Hall, M.J., Reid, J.E., Burbidge, L.A., Pruss, D., Deffenbaugh, A.M., Frye, C., Wenstrup, R.J., Ward, B.E., Scholl, T.A., and Noll, W.W. (2009). BRCA1 and BRCA2 mutations in women of different ethnicities undergoing testing for hereditary breast-ovarian cancer. Cancer *115*, 2222–2233.
19. Shaikh, T., Handorf, E.A., Meyer, J.E., Hall, M.J., and Esnaola, N.F. (2018). Mismatch repair deficiency testing in patients with colorectal cancer and nonadherence to testing guidelines in young adults. JAMA Oncol. *4*, e173580.
20. Cruz-Correa, M., Pérez-Mayoral, J., Dutil, J., Echenique, M., Mosquera, R., Rivera-Román, K., Umpierre, S., Rodriguez-Quilichini, S., Gonzalez-Pons, M., Olivera, M.I., Pardo, S.; and Puerto Rico Clinical Cancer Genetics Consortia (2017). Hereditary cancer syndromes in Latino populations: genetic characterization and surveillance guidelines. Hered. Cancer Clin. Pract. *15*, 3.
21. Jagsi, R., Griffith, K.A., Kurian, A.W., Morrow, M., Hamilton, A.S., Graff, J.J., Katz, S.J., and Hawley, S.T. (2015). Concerns about cancer risk and experiences with genetic testing in a diverse population of patients with breast cancer. J. Clin. Oncol. *33*, 1584–1591.
22. Rosenstock, I.M., Strecher, V.J., and Becker, M.H. (1988). Social learning theory and the health belief model. Health Educ. Q. *15*, 175–183.
23. Shiloh, S. (2006). Illness representations, self-regulation, and genetic counseling: a theoretical review. J. Genet. Couns. *15*, 325–337.
24. Rogers, R.W. (1983). Cognitive and physiological processes in fear appeals and attitude change: a revised theory of protection motivation. In Social Psychophysiology, J.T. Cacioppo and R.E. Petty, eds. (Guilford Press), pp. 153–176.
25. Christensen, K.D., Roberts, J.S., Zikmund-Fisher, B.J., Kardia, S.L., McBride, C.M., Linnenbringer, E., Green, R.C.; and REVEAL Study Group (2015). Associations between self-referral and health behavior responses to genetic risk information. Genome Med. *7*, 10.
26. Sweeny, K., Ghane, A., Legg, A.M., Huynh, H.P., and Andrews, S.E. (2014). Predictors of genetic testing decisions: a systematic review and critique of the literature. J. Genet. Couns. *23*, 263–288.
27. Sorlie, P.D., Avilés-Santa, L.M., Wassertheil-Smoller, S., Kaplan, R.C., Daviglus, M.L., Giachello, A.L., Schneiderman, N., Raij, L., Talavera, G., Allison, M., et al. (2010). Design and implementation of the hispanic community health study/study of Latinos. Ann. Epidemiol. *20*, 629–641.
28. Lavange, L.M., Kalsbeek, W.D., Sorlie, P.D., Avilés-Santa, L.M., Kaplan, R.C., Barnhart, J., Liu, K., Giachello, A., Lee, D.J., Ryan, J., et al. (2010). Sample design and cohort selection in the hispanic community health study/study of Latinos. Ann. Epidemiol. *20*, 642–649.
29. Nelson, D.E., Kreps, G.L., Hesse, B.W., Croyle, R.T., Willis, G., Arora, N.K., Rimer, B.K., Viswanath, K.V., Weinstein, N., and Alden, S. (2004). The health information national trends survey (HINTS): development, design, and dissemination. J. Health Commun. *9*, 443–460. discussion 481-444.
30. Vassy, J.L., Christensen, K.D., Slashinski, M.J., Lautenbach, D.M., Raghavan, S., Robinson, J.O., Blumenthal-Barby, J., Feuerman, L.Z., Lehmann, L.S., Murray, M.F., et al. (2015). 'Someday it will be the norm': physician perspectives on the utility of genome sequencing for patient care in the MedSeq Project. Per. Med. *12*, 23–32.
31. Krakow, M., Ratcliff, C.L., Hesse, B.W., and Greenberg-Worisek, A.J. (2017). Assessing genetic literacy awareness and knowledge gaps in the US population: results from the Health Information National Trends Survey. Public Health Genomics *20*, 343–348.
32. Mavaddat, N., Michailidou, K., Dennis, J., Lush, M., Fachal, L., Lee, A., Tyrer, J.P., Chen, T.H., Wang, Q., Bolla, M.K., et al. (2019). Polygenic risk scores for prediction of breast cancer and breast cancer subtypes. Am. J. Hum. Genet. *104*, 21–34.
33. Natarajan, P., Young, R., Stitziel, N.O., Padmanabhan, S., Baber, U., Mehran, R., Sartori, S., Fuster, V., Reilly, D.F., Butterworth, A., et al. (2017). Polygenic risk score identifies subgroup with higher burden of atherosclerosis and greater relative benefit from statin therapy in the primary prevention setting. Circulation *135*, 2091–2101.
34. Caraballo, P.J., Sutton, J.A., Giri, J., Wright, J.A., Nicholson, W.T., Kullo, I.J., Parkulo, M.A., Bielinski, S.J., and Moyer, A.M. (2020). Integrating pharmacogenomics into the electronic health record by implementing genomic indicators. J. Am. Med. Inform. Assoc. *27*, 154–158.
35. Hurle, B., Citrin, T., Jenkins, J.F., Kaphingst, K.A., Lamb, N., Roseman, J.E., and Bonham, V.L. (2013). What does it mean to be genomically literate?: national human genome research Institute meeting report. Genet. Med. *15*, 658–663.
36. Collins, F.S., and Varmus, H. (2015). A new initiative on precision medicine. N. Engl. J. Med. *372*, 793–795.
37. Mai, P.L., Vadaparampil, S.T., Breen, N., McNeel, T.S., Wideroff, L., and Graubard, B.I. (2014). Awareness of cancer susceptibility genetic testing: the 2000, 2005, and 2010 national health interview surveys. Am. J. Prev. Med. *46*, 440–448.
38. Pagán, J.A., Su, D., Li, L., Armstrong, K., and Asch, D.A. (2009). Racial and ethnic disparities in awareness of genetic testing for cancer risk. Am. J. Prev. Med. *37*, 524–530.
39. Heck, J.E., Franco, R., Jurkowski, J.M., and Sheinfeld Gorin, S. (2008). Awareness of genetic testing for cancer among United

States Hispanics: the role of acculturation. Community Genet. *11*, 36–42.
40. Salloum, R.G., George, T.J., Silver, N., Markham, M.J., Hall, J.M., Guo, Y., Bian, J., and Shenkman, E.A. (2018). Rural-urban and racial-ethnic differences in awareness of direct-to-consumer genetic testing. BMC Publ. Health *18*, 277.
41. González Burchard, E., Borrell, L.N., Choudhry, S., Naqvi, M., Tsai, H.J., Rodriguez-Santana, J.R., Chapela, R., Rogers, S.D., Mei, R., Rodriguez-Cintron, W., et al. (2005). Latino populations: a unique opportunity for the study of race, genetics, and social environment in epidemiological research. Am. J. Public Health *95*, 2161–2168.
42. Gonzalez, S., Strizich, G., Isasi, C.R., Hua, S., Comas, B., Sofer, T., Thyagarajan, B., Perreira, K.M., Talavera, G.A., Daviglus, M.L., et al. (2021). Consent for use of genetic data among US hispanics/latinos: results from the hispanic community health study/study of Latinos. Ethn. Dis. *31*, 547–558.
43. Hamilton, J.G., Shuk, E., Arniella, G., González, C.J., Gold, G.S., Gany, F., Robson, M.E., and Hay, J.L. (2016). Genetic testing awareness and attitudes among Latinos: exploring shared perceptions and gender-based differences. Public Health Genomics *19*, 34–46.
44. Landry, L.G., Ali, N., Williams, D.R., Rehm, H.L., and Bonham, V.L. (2018). Lack of diversity in genomic databases Is a barrier to translating precision medicine research into practice. Health Aff. *37*, 780–785.
45. Kim, I.E., Jr., and Sarkar, I.N. (2019). Racial representation disparity of population-level genomic sequencing efforts. Stud. Health Technol. Inform. *264*, 974–978.
46. Roberts, M.C., Mensah, G.A., and Khoury, M.J. (2019). Leveraging implementation science to address health disparities in genomic medicine: examples from the field. Ethn. Dis. *29*, 187–192.
47. Martin, A.R., Kanai, M., Kamatani, Y., Okada, Y., Neale, B.M., and Daly, M.J. (2019). Clinical use of current polygenic risk scores may exacerbate health disparities. Nat. Genet. *51*, 584–591.
48. Spratt, D.E., Chan, T., Waldron, L., Speers, C., Feng, F.Y., Ogunwobi, O.O., and Osborne, J.R. (2016). Racial/ethnic disparities in genomic sequencing. JAMA Oncol. *2*, 1070–1074.
49. The All of Us Research Program Investigators (2019). The “all of us” research Program. N. Engl. J. Med. *381*, 668–676.
50. Arafah, A., AlJawadi, M.H., Aldheefi, M., and Rehman, M.U. (2021). Attitude and awareness of public towards genetic testing in Riyadh, Saudi Arabia. Saudi J. Biol. Sci. *28*, 255–261.
51. Altaany, Z., Khabour, O.F., and Al-Taani, G. (2019). Knowledge, beliefs, and attitudes concerning genetic testing among young Jordanians. J. Multidiscip. Healthc. *12*, 1043–1048.
52. Hanson, T. (2020). SIENNA D2.5: Public Views on Genetics, Genomics and Gene Editing in 11 EU and Non-eu Countries (Zenodo). https://doi.org/10.5281/zenodo.4081155.

HGGA, Volume *4*

Supplemental information

Awareness and utilization of genetic testing among Hispanic and Latino adults living in the US: The Hispanic Community Health Study/Study of Latinos

Kurt D. Christensen, Mengran Zhang, Lauren N. Galbraith, Einat Granot-Hershkovitz, Sarah C. Nelson, Sara Gonzalez, Maria Argos, Krista M. Perreira, Martha L. Daviglus, Carmen R. Isasi, Jianwen Cai, Gregory A. Talavera, Carrie L. Blout Zawatsky, Robert C. Green, Rosario Isasi, Robert Kaplan, and Tamar Sofer

Awareness and Utilization of Genetic Testing among Hispanic/Latino Adults Living in the US: The Hispanic Community Health Study/Study of Latinos: Supplemental Information

Table of Contents

Figure S1. Spanish version of the genetic testing awareness survey. 2

Figure S2. English version of the genetic testing awareness survey. 4

Figure S3: Proportion of aware participants who were offered and used genetic tests. 6

Figure S4: Awareness, offers, and usage of genetic tests, stratified by gender. 7

Figure S5. Awareness of genetic tests, by demographic factors. 8

Table S1. Characteristics of survey participants. 10

Table S2. Logistic regression models for awareness of genetic tests, by site. 12

Table S3. Logistic regression models for awareness of genetic tests, by gender. 14

Table S4. Logistic regression models about awareness of specific types of genetic tests. 16

Table S5. Poisson regression models of perceived utility, by site. 18

Table S6. Poisson regression models of perceived utility, by gender. 20

Figure S1. Spanish version of the genetic testing awareness survey.

HCHS/SOL- Follow Up Interview

Genetic Testing Awareness, GTS

ID NUMBER:		FORM CODE: GTS VERSION: 1, 4/8/2019	Contact Occasion	0	1	Occurrence	0	1

ADMINISTRATIVE INFORMATION

0a. Completion Date: ☐☐/☐☐/☐☐☐☐ 0b. Staff ID: ☐☐☐

Instructions: Enter the answer given by the participant for each response. Use the CDART Notelog window to code 'Don't know/refused, Missing, etc.' for those questions that do not list these as an option.

INTRODUCTION: Los médicos utilizan estudios genéticos para analizar los genes de las personas por razones de salud.

1. ¿Ha oído usted hablar sobre una prueba genética para determinar el riesgo o la probabilidad de contraer una enfermedad en particular?

   No 0 ☐ **[Go to item 1c]** Sí 1 ☐ Rehusa contestar 9 ☐ **[Go to item 2]**

   1a. ¿Le han ofrecido alguna vez este tipo de prueba?

   No 0 ☐ **[Go to item 1c]** Sí 1 ☐ No sé 8 ☐**[Go to item 1c]** Rehusa contestar 9 ☐

   1b. ¿Le han hecho alguna vez este tipo de prueba?

   No 0 ☐ Sí 1 ☐ **[Go to item 2]** No sé 8 ☐ Rehusa contestar 9 ☐

   1c. Si se la ofrecieran, ¿estaría usted interesado(a) en hacerse este tipo de prueba?

   No 0 ☐ Sí 1 ☐ No estoy seguro(a)/depende 2 ☐ Rehusa contestar 9 ☐

2. ¿Ha oído usted hablar sobre una prueba genética para determinar la probabilidad de transmitir una enfermedad hereditaria a sus hijos?

   No 0 ☐ **[Go to item 2c]** Sí 1 ☐ Rehusa contestar 9 ☐ **[Go to item 3]**

   2a. ¿Le han ofrecido alguna vez este tipo de prueba?

   No 0 ☐ **[Go to item 2c]** Sí 1 ☐ No sé 8 ☐**[Go to item 2c]** Rehusa contestar 9 ☐

   2b. ¿Le han hecho alguna vez este tipo de prueba?

   No 0 ☐ Sí 1 ☐ **[Go to item 3]** No sé 8 ☐ Rehusa contestar 9 ☐

   2c. Si se la ofrecieran, ¿estaría usted interesado(a) en hacerse este tipo de prueba?

   No 0 ☐ Sí 1 ☐ No estoy seguro(a)/depende 2 ☐ Rehusa contestar 9 ☐

3. ¿Ha oído usted hablar sobre una prueba genética para determinar cómo se debe tratar una enfermedad después del diagnóstico?

   No 0 ☐ **[Go to item 3c]** Sí 1 ☐ Rehusa contestar 9 ☐ **[Go to item 4]**

   3a. ¿Le han ofrecido alguna vez este tipo de prueba?

   No 0 ☐ **[Go to item 3c]** Sí 1 ☐ No sé 8 ☐**[Go to item 3c]** Rehusa contestar 9 ☐

   3b. ¿Le han hecho alguna vez este tipo de prueba?

   No 0 ☐ Sí 1 ☐ **[Go to item 4]** No sé 8 ☐ Rehusa contestar 9 ☐

   3c. Si se la ofrecieran, ¿estaría usted interesado(a) en hacerse este tipo de prueba?

   No 0 ☐ Sí 1 ☐ No estoy seguro(a)/depende 2 ☐ Rehusa contestar 9 ☐

Figure S1 (continued).

ID NUMBER:									FORM CODE: GTS<br>VERSION: 1, 4/8/2019	Contact Occasion			Occurrence		

4. ¿Ha oído usted hablar sobre una prueba genética para determinar los medicamentos que pueden servirle o no a una persona (individuo)?

   No 0 ☐ **[Go to item 4c]** Sí 1 ☐ Rehusa contestar 9 ☐ **[Go to item 5]**

   4a. ¿Le han ofrecido alguna vez este tipo de prueba?

   No 0 ☐ **[Go to item 4c]** Sí 1 ☐ No sé 8 ☐**[Go to item 4c]** Rehusa contestar 9 ☐

   4b. ¿Le han hecho alguna vez este tipo de prueba?

   No 0 ☐ Sí 1 ☐ **[Go to item 5]** No sé 8 ☐ Rehusa contestar 9 ☐

   4c. Si se la ofrecieran, ¿estaría usted interesado(a) en hacerse este tipo de prueba?
   No 0 ☐ Sí 1 ☐ No estoy seguro(a)/depende 2 ☐ Rehusa contestar 9 ☐

5. En una escala del 1 al 10, donde 1 es "de ningún modo" y 10 es "absolutamente", ¿qué tan útil cree usted que es la prueba genética para controlar la salud de una persona? ☐☐

Figure S2. English version of the genetic testing awareness survey.

HCHS/SOL- Follow Up Interview

Genetic Testing Awareness, GTE

ID NUMBER:									FORM CODE: GTE VERSION: 1, 4/8/2019	Contact Occasion	0	1	Occurrence	0	1

ADMINISTRATIVE INFORMATION

0a. Completion Date: ☐☐/☐☐/☐☐☐☐ 0b. Staff ID: ☐☐☐

Instructions: Enter the answer given by the participant for each response. Use the CDART Notelog window to code 'Don't know/refused, Missing, etc.' for those questions that do not list these as an option.

INTRODUCTION: Doctors use genetic tests to analyze someone's genes for health reasons.

1. Have you heard of a genetic test to determine the risk or likelihood of getting a particular disease?

   No 0 ☐ **[Go to item 1c]** Yes 1 ☐ Refuse to answer 9 ☐**[Go to item 2]**

   1a. Have you ever been offered such test?

   No 0 ☐ **[Go to item 1c]** Yes 1 ☐ Don't know 8 ☐**[Go to item 1c]** Refuse to answer 9 ☐

   1b. Have you ever received this kind of test?

   No 0 ☐ Yes 1 ☐ **[Go to item 2]** Don't know 8 ☐ Refuse to answer 9 ☐

   1c. If offered to you, would you be interested in receiving this kind of test?

   No 0 ☐ Yes 1 ☐ Not sure / it depends 2 ☐ Refuse to answer 9 ☐

2. Have you heard of a genetic test to determine the likelihood of passing an inherited disease to your children?

   No 0 ☐ **[Go to item 2c]** Yes 1 ☐ Refuse to answer 9 ☐**[Go to item 3]**

   2a. Have you ever been offered such test?

   No 0 ☐ **[Go to item 2c]** Yes 1 ☐ Don't know 8 ☐**[Go to item 2c]** Refuse to answer 9 ☐

   2b. Have you ever received this kind of test?

   No 0 ☐ Yes 1 ☐ **[Go to item 3]** Don't know 8 ☐ Refuse to answer 9 ☐

   2c. If offered to you, would you be interested in receiving this kind of test?

   No 0 ☐ Yes 1 ☐ Not sure / it depends 2 ☐ Refuse to answer 9 ☐

3. Have you heard of a genetic test to determine how a disease should be treated after diagnosis?

   No 0 ☐ **[Go to item 3c]** Yes 1 ☐ Refuse to answer 9 ☐**[Go to item 4]**

   3a. Have you ever been offered such test?

   No 0 ☐ **[Go to item 3c]** Yes 1 ☐ Don't know 8 ☐**[Go to item 3c]** Refuse to answer 9 ☐

   3b. Have you ever received this kind of test?

   No 0 ☐ Yes 1 ☐ **[Go to item 4]** Don't know 8 ☐ Refuse to answer 9 ☐

   3c. If offered to you, would you be interested in receiving this kind of test?

   No 0 ☐ Yes 1 ☐ Not sure / it depends 2 ☐ Refuse to answer 9 ☐

GTE_Genetic Testing Awareness_English_20190408_FINAL 1 of 2

Figure S2 (continued).

ID NUMBER:									FORM CODE: GTE<br>VERSION: 1, 4/8/2019	Contact Occasion			Occurrence		

4. Have you heard of a genetic test to determine which drug(s) may or may not work for an individual?

   No 0 ☐ **[Go to item 4c]** Yes 1 ☐ Refuse to answer 9 ☐**[Go to item 5]**

   4a. Have you ever been offered such test?

   No 0 ☐ **[Go to item 4c]** Yes 1 ☐ Don't know 8 ☐**[Go to item 4c]** Refuse to answer 9 ☐

   4b. Have you ever received this kind of test?

   No 0 ☐ Yes 1 ☐ **[Go to item 5]** Don't know 8 ☐ Refuse to answer 9 ☐

   4c. If offered to you, would you be interested in receiving this kind of test?

   No 0 ☐ Yes 1 ☐ Not sure / it depends 2 ☐ Refuse to answer 9 ☐

5. On a scale of 1 to 10, where 1 is "not at all" and 10 is "extremely", how useful do you think genetic testing is for managing a person's health? ☐☐

Figure S3: Proportion of aware participants who were offered and used genetic tests.

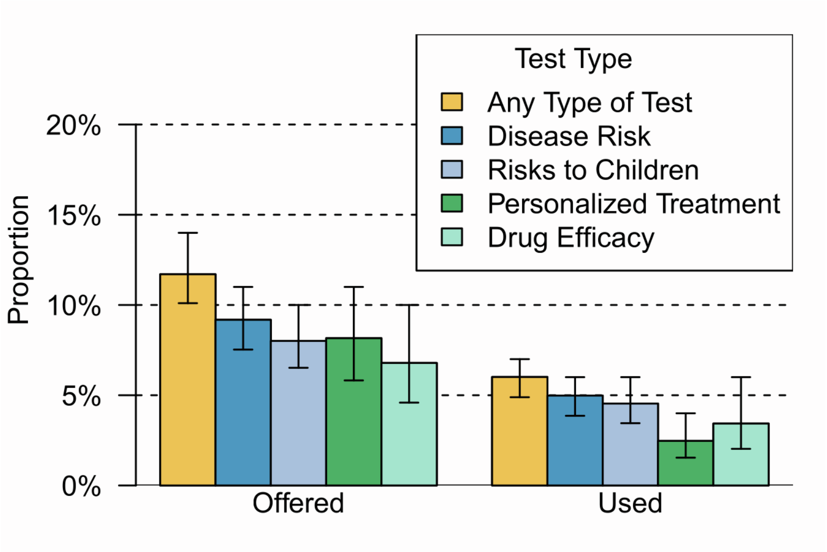

Bars represent estimates and 95% CIs of the proportions of participants who were aware of genetic tests that additionally reported ever being offered or using them.

Figure S4: Awareness, offers, and usage of genetic tests, stratified by gender.

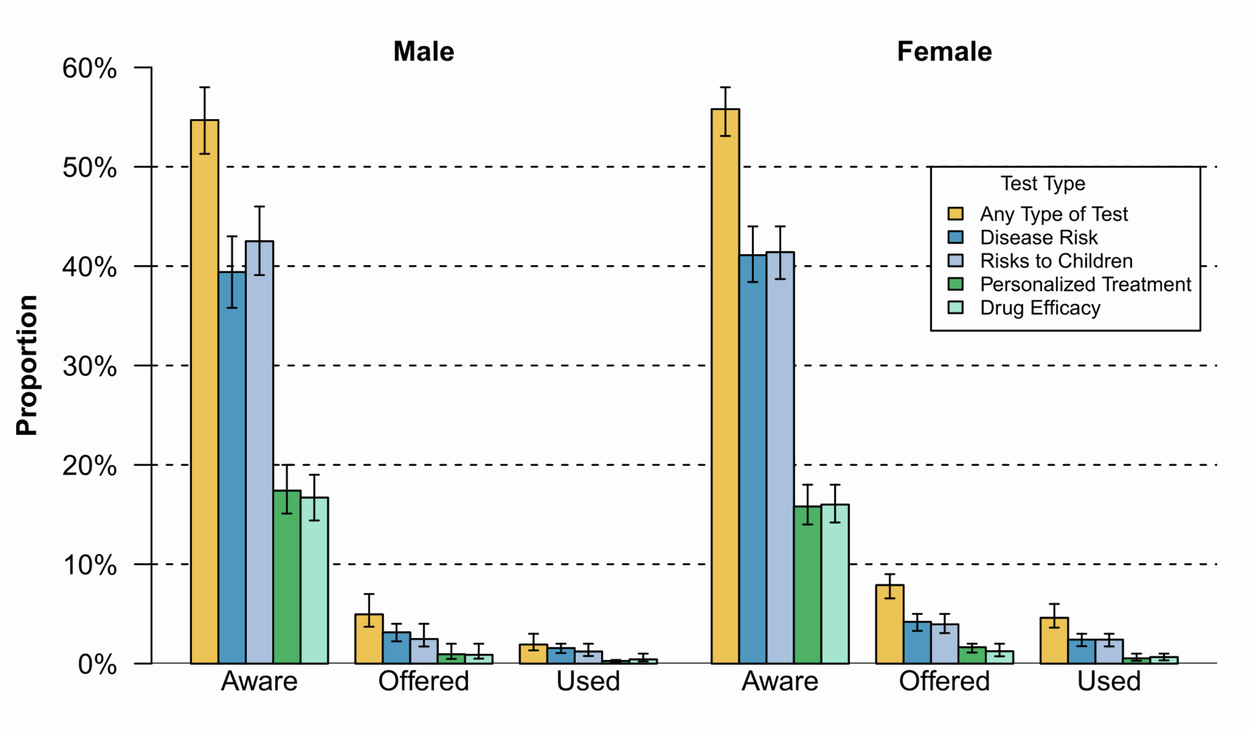

Bars represent estimates and 95% CIs of the proportions of participants who reported being aware of genetic tests, being offered them, or using them, stratified by gender.

Figure S5. Awareness of genetic tests, by demographic factors.

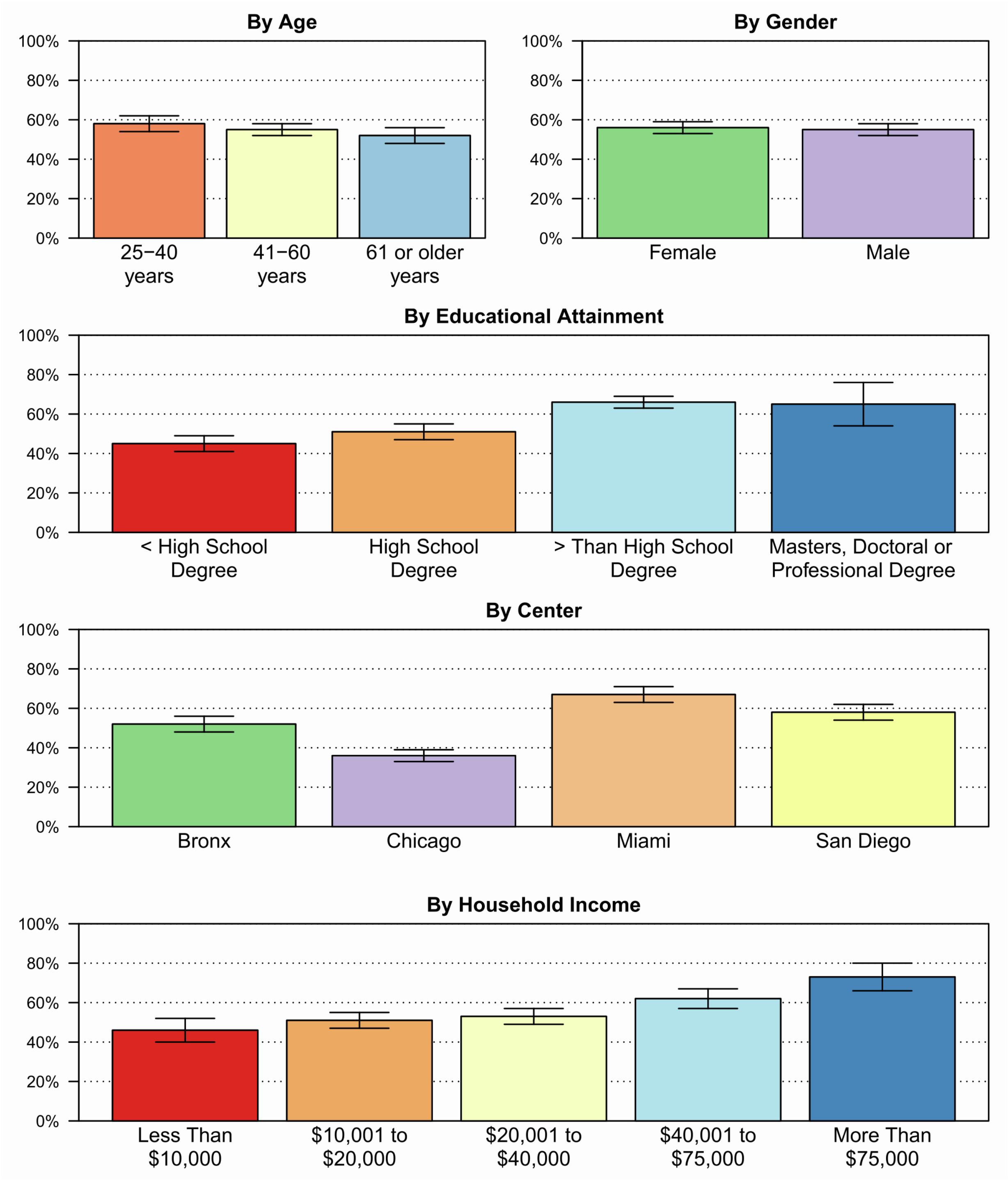

Figure S5 (continued)

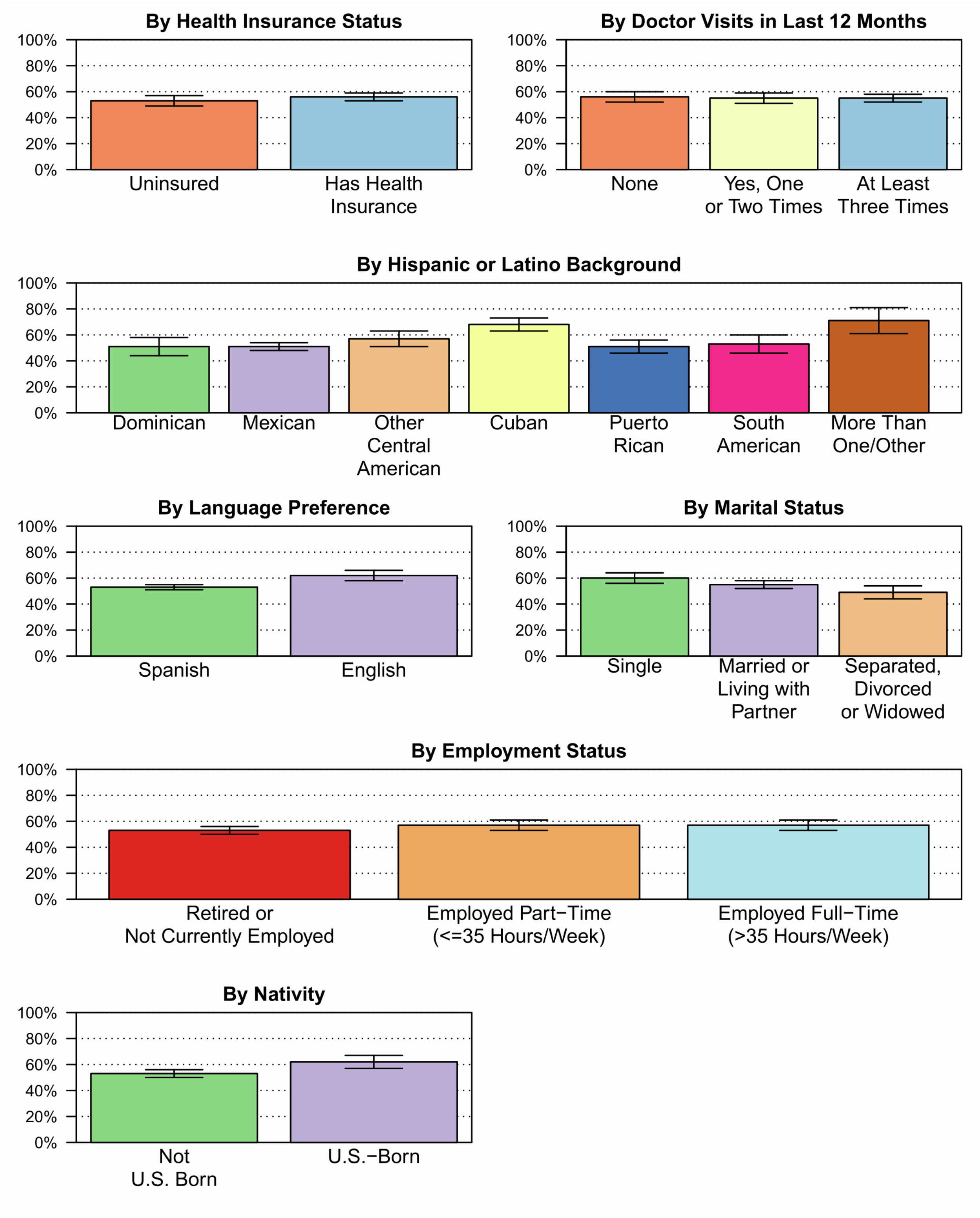

Bars represent estimates and 95% CIs of the proportions of participants who reported being aware of at least one type of genetic test, stratified by demographic factors of interest.

Table S1. Characteristics of survey participants.

Total N	Did not Complete Any Awareness Items (n=3,639)	Completed at Least One Awareness Item (n=5,769)	p	% Missing
Mean Age, years	46.31 (15.25)	45.81 (14.55)	0.081	0
Sex			0.023	0
Female	2249 (51.1)	3701 (53.3)		
Male	1390 (48.9)	2068 (46.7)		
Center			<0.001	0
Bronx	1357 (41.9)	1030 (21.7)		
Chicago	658 (9.0)	1950 (20.4)		
Miami	736 (30.4)	1065 (27.6)		
San Diego	888 (18.7)	1724 (30.4)		
Education			<0.001	0.5
Less than high school degree	1464 (36.9)	2004 (28.8)		
High school degree	946 (28.0)	1458 (26.9)		
Associate, bachelor, or vocational degree	1103 (32.9)	2078 (40.4)		
Masters, doctoral, professional degree	95 (2.1)	206 (4.0)		
Household Income			<0.001	3.1
Less than $10,000	486 (14.5)	613 (9.7)		
$10,001-$20,000	1057 (28.7)	1532 (25.7)		
$20,001-$40,000	1162 (32.8)	1999 (34.4)		
$40,001-$75,000	571 (17.2)	1039 (19.7)		
More than $75,000	214 (6.7)	441 (10.5)		
Health Insurance Status			0.690	0.7
Uninsured	1037 (30.9)	1686 (31.5)		
Has health insurance	2552 (69.1)	4068 (68.5)		
Doctor Visit in Last 12 Months			0.824	1.8

No	944 (31.1)	1521 (32.0)		
Yes, one or two times	1113 (30.9)	1820 (30.2)		
Yes, at least three times	1484 (38.0)	2352 (37.8)		
Hispanic or Latino background			<0.001	0.5
Dominican	449 (13.6)	413 (8.1)		
Mexican	1344 (29.4)	2753 (42.4)		
Central American	332 (7.7)	575 (7.0)		
Cuban	426 (21.2)	593 (19.2)		
Puerto Rican	717 (19.1)	820 (14.4)		
South American	228 (4.7)	424 (5.2)		
More than one/other	112 (4.4)	172 (3.7)		
Language Preference			<0.001	0
Spanish	2859 (73.2)	4731 (75.7)		
English	780 (26.8)	1038 (24.3)		
Marital Status			<0.001	0.3
Single	906 (33.1)	1234 (29.0)		
Married or living with partner	1935 (48.1)	3288 (53.5)		
Separated, divorced or widowed	777 (18.8)	1240 (17.5)		
Employment Status			<0.001	0.7
Retired/not currently employed	1660 (44.5)	2362 (37.4)		
Employed part-time (≤35 hours/week)	730 (20.1)	1318 (22.2)		
Employed full-time (>35 hours/week)	1199 (35.3)	2071 (40.4)		
Nativity				
Not U.S. Born	2969 (77.0)	4835 (77.5)	0.021	0.5
U.S.-Born	643 (23.0)	918 (22.5)		

The table summarizes characteristics of HCHS/SOL participants who participated in the annual follow-up survey. Analyses compare the characteristics of individuals who completed at least one genetic testing awareness item against characteristics of individuals who did not.

Table S2. Logistic regression models for awareness of genetic tests, by site.

	Bronx	Chicago	Miami	San Diego
**Term**	**OR (95% CI)**	**OR (95% CI)**	**OR (95% CI)**	**OR (95% CI)**
Age, years (ref: 40 and younger)				
41-60	0.93 (0.60-1.43)	1.07 (0.81-1.41)	0.89 (0.51-1.54)	1.14 (0.76-1.71)
61 or older	1.34 (0.77-2.33)	0.94 (0.60-1.46)	0.66 (0.34-1.28)	0.96 (0.47-1.94)
Male sex (ref: Female)	0.81 (0.54-1.21)	1.01 (0.76-1.35)	0.86 (0.60-1.22)	0.87 (0.60-1.25)
Education, years (ref: less than high school degree)				
High school degree	0.76 (0.47-1.21)	1.61 (1.16-2.22)*	1.16 (0.68-1.99)	1.02 (0.65-1.58)
Associate, bachelor, or vocational degree	1.90 (1.23-2.94)*	2.26 (1.56-3.27)**	1.44 (0.90-2.29)	1.70 (1.10-2.64)
Masters, doctoral, professional degree	1.46 (0.46-4.65)	3.17 (1.29-7.77)	1.04 (0.49-2.20)	2.80 (1.37-5.74)*
Income (ref: Less than $10,000)				
$10,001-$20,000	1.46 (0.86-2.49)	0.89 (0.51-1.55)	1.24 (0.69-2.24)	0.68 (0.36-1.28)
$20,001-$40,000	1.39 (0.73-2.64)	0.97 (0.58-1.62)	1.09 (0.60-1.98)	1.11 (0.61-2.03)
$40,001-$75,000	1.54 (0.71-3.31)	0.98 (0.54-1.77)	2.40 (1.11-5.22)	1.73 (0.86-3.48)
More than $75,000	1.60 (0.75-3.43)	1.60 (0.79-3.24)	1.87 (0.78-4.53)	3.24 (1.30-8.08)
Health Insurance Status (ref: None)	0.81 (0.41-1.57)	1.19 (0.85-1.67)	0.92 (0.63-1.33)	1.24 (0.82-1.86)
Doctor Visit in Last 12 Months (ref: none)				
Yes, one or two times	1.08 (0.57-2.03)	1.32 (0.93-1.88)	1.02 (0.65-1.58)	0.86 (0.57-1.30)
At least three times	1.20 (0.64-2.27)	1.37 (0.97-1.92)	0.74 (0.42-1.29)	1.00 (0.69-1.45)

Hispanic or Latino Background (ref: Dominican)				
Mexican	1.07 (0.49-2.33)	0.68 (0.24-1.92)	0.16 (0.02-1.24)	NA[†]
Central American	1.03 (0.45-2.35)	0.79 (0.25-2.47)	0.77 (0.28-2.10)	NA[†]
Cuban	4.02 (0.69-23.40)	0.77 (0.21-2.82)	0.96 (0.34-2.65)	NA[†]
Puerto Rican	1.08 (0.68-1.70)	0.62 (0.22-1.78)	1.14 (0.24-5.48)	NA[†]
South American	1.57 (0.71-3.45)	0.47 (0.16-1.33)	0.68 (0.21-2.20)	NA[†]
More than one/other	1.36 (0.52-3.55)	1.32 (0.35-4.98)	2.99 (0.58-15.40)	NA[†]
English Language Preference (ref: Spanish)	0.91 (0.53-1.56)	1.00 (0.63-1.59)	1.98 (0.72-5.39)	1.65 (1.10-2.48)
Marital Status (ref: Single)				
Married or living with partner	0.69 (0.47-1.02)	1.34 (0.99-1.82)	0.73 (0.44-1.22)	0.89 (0.57-1.40)
Separated, divorced or widowed	0.48 (0.27-0.85)	1.41 (0.89-2.23)	0.66 (0.37-1.20)	0.63 (0.36-1.12)
Employment Status (ref: Retired/not currently employed)				
Employed part-time (≤35 hours/week)	1.51 (0.88-2.59)	0.97 (0.62-1.53)	0.80 (0.49-1.31)	1.17 (0.76-1.78)
Employed full-time (>35 hours/week)	1.16 (0.69-1.93)	0.93 (0.66-1.31)	0.91 (0.58-1.44)	0.91 (0.60-1.39)
U.S.-born (ref: Not U.S.-born)	1.26 (0.71-2.25)	1.11 (0.67-1.84)	1.49 (0.66-3.38)	0.95 (0.61-1.48)

* p<0.01 ** p<0.001

The table summarizes logistic regression analyses that examined associations between participant characteristics and the likelihood of reporting awareness of at least one type of genetic test, with separate models for each HCHS/SOL site of enrollment. Estimates represent odds ratios with 95% confidence intervals, and shows the increase in odds of reporting awareness relative to the reference category. Analyses included all covariates jointly, and were weighted to generate estimates valid for the HCHS/SOL population.

[†] **Results by Hispanic or Latino background at the San Diego site are omitted because 94% self-reported Mexican heritage.**

Table S3. Logistic regression models for awareness of genetic tests, by gender.

	Women	Men
**Term**	**OR (95% CI)**	**OR (95% CI)**
Age, years (ref: 40 and younger)		
41-60	1.15 (0.87-1.52)	0.86 (0.61-1.22)
61 or older	1.16 (0.82-1.63)	0.69 (0.42-1.14)
Education, years (ref: less than high school degree)		
High school degree	1.03 (0.78-1.35)	1.05 (0.72-1.53)
Associate, bachelor, or vocational degree	1.50 (1.13-1.99)*	1.95 (1.36-2.80)**
Masters, doctoral, professional degree	1.10 (0.61-1.98)	2.07 (0.97-4.39)
Center (ref: Bronx)		
Chicago	0.64 (0.43-0.97)	0.44 (0.26-0.75)*
Miami	1.77 (1.09-2.89)	1.82 (0.98-3.37)
San Diego	1.47 (0.88-2.45)	0.92 (0.48-1.76)
Income (ref: Less than $10,000)		
$10,001-$20,000	1.16 (0.83-1.61)	1.17 (0.63-2.18)
$20,001-$40,000	1.52 (1.11-2.08)*	0.97 (0.52-1.81)
$40,001-$75,000	2.15 (1.42-3.27)**	1.44 (0.76-2.71)
More than $75,000	3.76 (2.20-6.42)**	1.93 (0.95-3.93)
Health Insurance Status (ref: None)	0.93 (0.72-1.20)	1.10 (0.77-1.55)
Doctor Visit in Last 12 Months (ref: none)		
Yes, one or two times	1.03 (0.75-1.43)	0.98 (0.69-1.39)
At least three times	1.06 (0.78-1.45)	0.95 (0.65-1.39)
Hispanic or Latino Background (ref: Dominican)		
Mexican	0.57 (0.32-1.03)	2.04 (0.93-4.47)
Other Central American	0.99 (0.57-1.75)	1.28 (0.57-2.90)
Cuban	0.90 (0.48-1.67)	1.90 (0.78-4.67)
Puerto Rican	0.69 (0.43-1.11)	1.61 (0.80-3.23)

South American	0.86 (0.46-1.62)	1.16 (0.54-2.48)
More than one/other	1.09 (0.44-2.67)	2.27 (0.91-5.70)
English Language Preference (ref: Spanish)	1.27 (0.89-1.80)	1.06 (0.72-1.55)
Marital Status (ref: Single)		
Married or living with partner	0.78 (0.58-1.05)	0.95 (0.68-1.33)
Separated, divorced or widowed	0.66 (0.47-0.95)	0.77 (0.48-1.23)
Employment Status (ref: Retired/not currently employed)		
Employed part-time (≤35 hours/week)	1.02 (0.77-1.36)	1.15 (0.72-1.82)
Employed full-time (>35 hours/week)	1.04 (0.79-1.38)	0.86 (0.58-1.27)
U.S.-born (ref: Not U.S.-born)	1.34 (0.90-1.98)	1.02 (0.65-1.58)

* p<0.01

** p<0.001

The table summarizes logistic regression analyses that examined associations between participant characteristics and the likelihood of reporting awareness of at least one type of genetic test. Estimates represent odds ratios with 95% confidence intervals, and shows the increase in odds of reporting awareness relative to the reference category. Analyses included all covariates jointly, and were weighted to generate estimates valid for the HCHS/SOL population.

Table S4. Logistic regression models about awareness of specific types of genetic tests.

Term	Disease Risk OR (95%CI)	Risks to Children OR (95%CI)	Personalized Treatment OR (95%CI)	Drug Efficacy OR (95%CI)
Age, years (ref: 40 and younger)				
41-60	0.90 (0.71-1.16)	0.89 (0.71-1.12)	1.47 (1.04-2.09)	1.23 (0.90-1.68)
61 or older	0.96 (0.71-1.28)	0.78 (0.59-1.04)	1.34 (0.90-1.99)	1.16 (0.80-1.70)
Male sex (ref: Female)	0.82 (0.67-1.00)	0.91 (0.74-1.11)	1.15 (0.91-1.45)	0.98 (0.77-1.24)
Education, years (ref: less than high school degree)				
High school degree	1.09 (0.85-1.40)	1.14 (0.88-1.48)	1.20 (0.87-1.66)	1.38 (1.01-1.89)
Associate, bachelor, or vocational degree	1.70 (1.34-2.17)**	1.71 (1.36-2.16)**	1.78 (1.30-2.43)**	1.81 (1.35-2.42)**
Masters, doctoral, professional degree	2.01 (1.20-3.38)*	1.93 (1.17-3.19)	2.45 (1.42-4.22)*	3.01 (1.86-4.85)**
Center (ref: Bronx)				
Chicago	0.87 (0.63-1.20)	0.74 (0.51-1.06)	0.94 (0.62-1.43)	1.21 (0.81-1.80)
Miami	3.02 (2.11-4.31)**	1.39 (0.92-2.11)	1.15 (0.74-1.80)	1.17 (0.73-1.87)
San Diego	1.50 (1.03-2.21)	1.19 (0.75-1.89)	0.82 (0.49-1.39)	1.34 (0.84-2.14)
Income (ref: Less than $10,000)				
$10,001-$20,000	1.17 (0.83-1.64)	1.04 (0.76-1.43)	0.97 (0.63-1.49)	1.00 (0.65-1.52)
$20,001-$40,000	1.18 (0.85-1.65)	1.12 (0.81-1.57)	0.89 (0.58-1.34)	0.84 (0.56-1.26)
$40,001-$75,000	1.81 (1.22-2.68)*	1.55 (1.07-2.26)	1.11 (0.70-1.75)	1.02 (0.64-1.62)
More than $75,000	3.44 (2.15-5.51)**	1.84 (1.18-2.85)*	1.55 (0.89-2.72)	1.33 (0.78-2.26)
Has health insurance (ref: does not)	1.05 (0.83-1.33)	0.98 (0.79-1.22)	1.21 (0.93-1.57)	1.03 (0.78-1.36)

Doctor visit (ref: none)				
Yes, one or two times	1.08 (0.85-1.39)	0.98 (0.77-1.24)	0.90 (0.69-1.18)	1.01 (0.76-1.33)
At least three times	0.94 (0.73-1.23)	0.95 (0.74-1.22)	0.86 (0.66-1.13)	0.91 (0.68-1.23)
Heritage (ref: Dominican)				
Mexican	0.65 (0.41-1.03)	0.90 (0.55-1.46)	1.04 (0.57-1.88)	1.09 (0.62-1.93)
Central American	0.64 (0.41-1.00)	1.15 (0.71-1.86)	0.75 (0.42-1.36)	1.01 (0.56-1.82)
Cuban	0.90 (0.56-1.44)	1.28 (0.75-2.17)	0.80 (0.45-1.41)	1.00 (0.53-1.87)
Puerto Rican	0.86 (0.56-1.34)	0.87 (0.60-1.27)	0.90 (0.54-1.52)	1.03 (0.62-1.70)
South American	0.84 (0.50-1.39)	0.98 (0.63-1.52)	0.61 (0.34-1.11)	0.78 (0.42-1.48)
More than one/other	1.02 (0.57-1.83)	1.55 (0.85-2.84)	1.44 (0.72-2.88)	1.52 (0.76-3.06)
English Language Preference (ref: Spanish)	1.25 (0.92-1.69)	1.13 (0.86-1.48)	1.38 (0.97-1.95)	1.39 (1.03-1.87)
Marital Status (ref: Single)				
Separated, divorced or widowed	0.84 (0.65-1.08)	0.91 (0.73-1.13)	1.16 (0.88-1.51)	1.03 (0.78-1.36)
Married or Living with Partner	0.81 (0.60-1.09)	0.78 (0.58-1.05)	0.91 (0.65-1.28)	0.67 (0.47-0.96)
Employment Status (ref: Retired/not currently employed)				
Employed full-time (>35 hours/week)	1.15 (0.89-1.48)	1.05 (0.82-1.34)	0.91 (0.66-1.26)	0.86 (0.62-1.19)
Employed part-time (≤35 hours/week)	0.98 (0.76-1.26)	1.10 (0.88-1.38)	0.67 (0.50-0.91)	0.79 (0.58-1.08)
U.S.-born (ref: Not U.S.-born)	1.05 (0.76-1.45)	1.11 (0.83-1.47)	1.04 (0.72-1.49)	0.93 (0.67-1.29)

* p<0.01 ** p<0.001

The table summarizes logistic regression analyses that examined associations between participant characteristics and the likelihood of reporting awareness of specific genetic tests. Estimates represent odds ratios with 95% confidence intervals, and shows the increase in odds of reporting awareness relative to the reference category. Analyses included all covariates jointly, and were weighted to generate estimates valid for the HCHS/SOL population.

Table S5. Poisson regression models of perceived utility, by site.

	Bronx	Chicago	Miami	San Diego
**Term**	**Estimate (95% CI)**	**Estimate (95% CI)**	**Estimate (95% CI)**	**Estimate (95% CI)**
Age, years (ref: 40 and younger)				
41-60	1.01 (0.94-1.08)	1.01 (0.96-1.05)	1.05 (1.01-1.09)	1.03 (0.98-1.09)
61 or older	0.91 (0.83-0.99)	0.95 (0.89-1.01)	1.05 (1.00-1.09)	1.01 (0.95-1.06)
Male sex (ref: Female)	0.97 (0.91-1.03)	1.00 (0.97-1.04)	0.99 (0.96-1.02)	0.97 (0.94-1.00)
Education, years (ref: less than high school degree)				
High school degree	1.04 (0.96-1.13)	1.01 (0.97-1.06)	0.99 (0.94-1.03)	1.00 (0.95-1.04)
Associate, bachelor, or vocational degree	1.08 (1.02-1.14)*	1.02 (0.97-1.07)	1.02 (0.97-1.06)	1.00 (0.95-1.05)
Masters, doctoral, professional degree	1.03 (0.90-1.18)	1.03 (0.95-1.11)	0.93 (0.85-1.01)	0.94 (0.86-1.04)
Income (ref: Less than $10,000)				
$10,001-$20,000	1.03 (0.94-1.13)	1.04 (0.97-1.10)	0.99 (0.93-1.06)	0.97 (0.90-1.05)
$20,001-$40,000	1.02 (0.92-1.13)	0.98 (0.93-1.04)	1.03 (0.98-1.08)	0.97 (0.90-1.05)
$40,001-$75,000	1.06 (0.96-1.18)	0.98 (0.91-1.06)	1.03 (0.98-1.08)	1.00 (0.92-1.09)
More than $75,000	0.93 (0.83-1.05)	0.93 (0.84-1.04)	1.01 (0.92-1.09)	0.99 (0.89-1.09)
Health Insurance Status (ref: None)	1.07 (0.98-1.16)	1.01 (0.97-1.05)	0.98 (0.95-1.01)	0.99 (0.95-1.03)
Doctor Visit in Last 12 Months (ref: none)				
Yes, one or two times	0.95 (0.87-1.03)	1.04 (1.00-1.08)	1.01 (0.99-1.04)	1.03 (0.98-1.08)
At least three times	1.00 (0.93-1.08)	1.02 (0.97-1.07)	0.97 (0.95-1.00)	1.02 (0.97-1.07)

Hispanic or Latino Background (ref: Dominican)				
Mexican	1.00 (0.90-1.11)	1.09 (0.87-1.36)	0.89 (0.74-1.07)	0.86 (0.81-0.91)**
Other Central American	1.05 (0.99-1.11)	1.11 (0.88-1.39)	0.93 (0.87-1.00)	0.88 (0.76-1.02)
Cuban	0.89 (0.68-1.17)	1.16 (0.90-1.48)	0.97 (0.91-1.04)	0.92 (0.76-1.11)
Puerto Rican	1.01 (0.95-1.06)	1.14 (0.91-1.43)	0.98 (0.88-1.08)	0.88 (0.76-1.02)
South American	1.03 (0.96-1.11)	1.11 (0.88-1.41)	0.91 (0.86-0.97)*	0.88 (0.73-1.05)
More than one/other	1.07 (0.96-1.18)	1.03 (0.81-1.32)	1.00 (0.92-1.07)	0.79 (0.71-0.88)**
English Language Preference (ref: Spanish)	0.94 (0.89-0.99)	0.97 (0.88-1.06)	1.01 (0.93-1.10)	0.94 (0.89-0.99)
Marital Status (ref: Single)				
Married or living with partner	1.06 (0.99-1.15)	0.95 (0.89-1.01)	1.00 (0.96-1.04)	1.00 (0.96-1.05)
Separated, divorced or widowed	1.09 (1.01-1.17)	0.98 (0.91-1.06)	0.99 (0.95-1.03)	1.01 (0.96-1.07)
Employment Status (ref: Retired/not currently employed)				
Employed part-time (≤35 hours/week)	1.05 (1.00-1.11)	0.99 (0.95-1.04)	1.01 (0.97-1.06)	1.01 (0.97-1.06)
Employed full-time (>35 hours/week)	1.01 (0.94-1.08)	0.99 (0.95-1.04)	1.00 (0.97-1.04)	0.98 (0.94-1.03)
U.S.-born (ref: Not U.S.-born)	0.96 (0.90-1.03)	0.90 (0.83-0.96)*	0.93 (0.87-0.98)	0.97 (0.93-1.01)

* $p<0.01$ ** $p<0.001$

The table summarizes Poisson regression analyses that examined associations between participant characteristics and the perceived utility of genetic testing, rated on a 1-10 scale, with separate models for each study site. Estimates represent exponentiated coefficients, and show the increase in perceived utility scores relative to the reference category. Analyses included all covariates jointly, and were weighted to generate estimates valid for the HCHS/SOL population.

Table S6. Poisson regression models of perceived utility, by gender.

Term	Women<br>Estimate (95% CI)	Men<br>Estimate (95% CI)
Age, years (ref: 40 and younger)		
41-60	1.03 (1.00-1.07)	1.01 (0.96-1.07)
61 or older	0.99 (0.95-1.03)	0.97 (0.91-1.03)
Education, years (ref: less than high school degree)		
High school degree	1.03 (1.00-1.06)	0.99 (0.94-1.05)
Associate, bachelor, or vocational degree	1.02 (0.99-1.05)	1.05 (1.00-1.09)
Masters, doctoral, professional degree	1.01 (0.94-1.08)	0.93 (0.85-1.01)
Center (ref: Bronx)		
Chicago	0.97 (0.92-1.02)	1.06 (0.98-1.15)
Miami	1.04 (0.99-1.10)	1.10 (1.02-1.18)
San Diego	0.99 (0.93-1.04)	1.08 (0.98-1.19)
Income (ref: Less than $10,000)		
$10,001-$20,000	1.05 (0.99-1.10)	0.95 (0.86-1.04)
$20,001-$40,000	1.04 (0.99-1.10)	0.95 (0.87-1.03)
$40,001-$75,000	1.06 (1.00-1.12)	0.98 (0.91-1.06)
More than $75,000	1.04 (0.97-1.12)	0.92 (0.84-1.00)
Health Insurance Status (ref: None)	1.01 (0.98-1.04)	0.98 (0.94-1.02)
Doctor Visit in Last 12 Months (ref: none)		
Yes, one or two times	1.00 (0.97-1.04)	1.01 (0.97-1.05)
At least three times	0.98 (0.95-1.02)	1.04 (1.00-1.09)
Hispanic or Latino Background(ref: Dominican)		
Mexican	1.05 (0.99-1.12)	0.92 (0.82-1.03)
Other Central American	1.02 (0.97-1.08)	0.97 (0.90-1.05)
Cuban	1.04 (0.98-1.11)	0.99 (0.91-1.09)

Puerto Rican	1.00 (0.93-1.07)	0.97 (0.89-1.06)
South American	1.02 (0.97-1.08)	0.94 (0.85-1.04)
More than one/other	1.06 (0.98-1.14)	0.89 (0.80-0.99)
English Language Preference (ref: Spanish)	0.97 (0.93-1.01)	0.93 (0.88-0.98)*
Marital Status (ref: Single)		
Married or living with partner	0.99 (0.96-1.02)	1.05 (1.01-1.10)
Separated, divorced or widowed	1.02 (0.98-1.05)	1.03 (0.97-1.09)
Employment Status (ref: Retired/not currently employed)		
Employed part-time (≤35 hours/week)	1.01 (0.98-1.04)	1.04 (0.99-1.09)
Employed full-time (>35 hours/week)	0.99 (0.97-1.02)	1.01 (0.97-1.05)
U.S.-born (ref: Not U.S.-born)	0.95 (0.91-0.99)	0.97 (0.92-1.02)

The table summarizes Poisson regression analyses that examined associations between participant characteristics and the perceived utility of genetic testing, rated on a 1-10 scale, with separate models for each gender. Estimates represent exponentiated coefficients, and show the relative increase in perceived efficacy scores relative to the reference category. Analyses included all covariates jointly, and were weighted to generate estimates valid for the HCHS/SOL target population. No findings were statistically significant at p<0.01.
